# Use of Imaging Modalities in Real Life: Impact on Visual Acuity Outcomes of Ranibizumab Treatment for Neovascular Age-Related Macular Degeneration in Germany

**DOI:** 10.1155/2020/8024258

**Published:** 2020-07-16

**Authors:** Joachim Wachtlin, Georg Spital, Steffen Schmitz-Valckenberg, Sandra Liakopoulos, Jessica Vögeler, Bettina Müller, Focke Ziemssen, Ocean study group

**Affiliations:** ^1^Department of Ophthalmology, Sankt-Gertrauden Krankenhaus, Berlin, Germany; ^2^MHB Medizinische Hochschule Brandenburg, Neuruppin, Germany; ^3^Eye Center, St. Franziskus-Hospital, Muenster, Germany; ^4^Department of Ophthalmology, University of Bonn, Bonn, Germany; ^5^John A. Moran Eye Center, University of Utah, Salt Lake City, UT, USA; ^6^Department of Ophthalmology, Faculty of Medicine and University Hospital of Cologne, Cologne, Germany; ^7^Novartis Pharma GmbH, Nuremberg, Germany; ^8^Centre for Ophthalmology, Eberhard-Karls-University Tuebingen, Tuebingen, Germany

## Abstract

**Background:**

To date, there are limited prospective real-world data on the impact of optical coherence tomography (OCT) diagnostics on treatment outcomes in neovascular age-related macular degeneration (nAMD). Therefore, the prospective, noninterventional OCEAN study (NCT02194803) evaluated the use of OCT imaging and its impact on functional outcomes in Germany.

**Methods:**

The use of OCT imaging for treatment decisions was documented in nAMD patients receiving intravitreal ranibizumab injections at 347 study centres. Best-corrected visual acuity (BCVA) testing and treatment were performed according to routine clinical practice and documented over 24 months.

**Results:**

The majority of the 3,631 nAMD patients (59.6%) received a combination of OCT and fluorescein angiography imaging within the first 6 months. Over the remaining study course, this combination was used infrequently (range: 7.6% to 13.4%) and continually decreased over time; most patients received only OCT examinations (range: 48.9% to 52.5%; median: 3 within 12 months and 4 within 24 months). Subgroups according to the number of OCT examinations (≤4, rarely OCT examined; 5–8, moderately OCT examined; ≥8, well monitored) were associated with different treatment frequencies and outcomes: Rarely OCT-examined patients had received a median of 4 injections (range: 1–19) at 24 months; well-monitored patients had received a median of 8 injections (range: 1–21) at 24 months. Rarely OCT-examined patients had a mean change of BCVA of −0.3 letters (±26.1) at 24 months (*n* = 165); well-monitored patients showed a change of +2.0 letters (±20.8) at 24 months (*n* = 249). Time-to-response was greater for rarely examined than well-monitored patients, while duration-of-response was similar.

**Conclusion:**

Low number of visits as well as high number of treatment decisions without the use of OCT may contribute to undertreatment and poorer functional outcomes in patients undergoing ranibizumab treatment for nAMD in Germany. One potential reason for this could be that OCT was not covered by insurance for all patients during the study.

## 1. Introduction

The association between neovascular age-related macular degeneration (nAMD) and vascular endothelial growth factor (VEGF) is well-documented [1, 2], with clinical trials showing that anti-VEGF injections improve visual acuity and prevent vision loss over time [3]. At the time of the study, the available anti-VEGF compounds for the treatment of nAMD included ranibizumab (Lucentis^®^, Novartis Pharma), aflibercept (Eylea®, Bayer Healthcare), and the off-label use of bevacizumab (Avastin®, Genentech) [4]. Most scientific societies recommend an initial upload of three anti-VEGF injections and follow-up, where the decision to retreat essentially depends on disease activity criteria mainly based on optical coherence tomography (OCT) [5].

For decades, fundoscopy and fluorescein angiography (FA) have been used to detect the presence of active macular neovascularization. OCT has been proven to add valuable and essential information about disease activity. Retinal thickness and subretinal fluid assessed by OCT examinations have been shown in previous studies to reflect the response to a chosen treatment [3, 6–8]. The activity of nAMD and subsequent treatment decisions are mainly based on OCT detection of presence of subretinal fluid (SRF), intraretinal fluid (IRF, cystoid spaces or diffuse retinal thickening), increase in pigment epithelial detachments (PEDs), and new hemorrhage [9, 10].

Nonadherence to monthly reassessments has been shown to be associated with fewer anti-VEGF injections in real-world practice [11, 12], while low annual number of treatments has been reported to have immediate impact on visual outcomes [13, 14], indicating the great importance of regularly scheduled visits to avoid undertreatment [15]. Although physicians applying this treatment are required to undergo training before they are allowed to inject—in contrast to oral therapy or eye drops—little is known about the adherence to guidelines regarding imaging modalities of follow-up recommendations in Germany [5]. To demonstrate OCT effectiveness in a clinical environment, the OCTAVE trial was initiated [16]. However, the study (NCT01780935) was terminated prematurely, due to the judgment that further evidence on the use of OCT was not needed. Other trials provided indirect hints that individualized treatment regimens such as as-needed treatment or treat and extend to achieve comparable outcomes [17–20]. Despite limited evidence from RCTs proving the use of OCT within the diagnostic-therapeutic chain, general consensus from synthesized evidence of systematic reviews and meta-analyses confirm the need for, and effectiveness of, OCT imaging. Evaluation of active macular neovascularization activity on OCT is challenging in routine clinical practice, due to the complex morphological changes including PEDs, subretinal hyperreflective material (SHRM), outer retinal tubulations, and macular atrophy, among others [21]. Also, OCT examinations have a higher false-positive rate when compared to FA, for example, as cystoid spaces may be present in the eyes without leakage on FA, thereby representing chronic degenerative changes rather than signs for active macular neovascularization activity [22]. Despite some disadvantages of OCT, the large majority of as-needed treatment decisions in RCTs have been based on OCT examinations (over 95% in the CATT study [3] and over 97% in the IVAN study [23]).

The aim of this study was to describe the use of OCT imaging and their impact on the results of treatment with anti-VEGF injections (ranibizumab, 0.5 mg) for nAMD patients in routine clinical practice.

## 2. Materials and Methods

### 2.1. Study Design

Data for the current report were obtained from the nAMD subset of patients from the prospective, multicentre, noninterventional OCEAN study (**O**bservation of treatment patterns with Lu**CE**ntis and real life ophthalmic monitoring, including optional OCT in **A**pproved i**N**dications, NCT02194803). The OCEAN study examined the use of intravitreal ranibizumab in adults with active nAMD or with visual impairment due to diabetic macular edema (DME), due to macular edema following retinal vein occlusion (RVO) or due to choroidal neovascularization following pathologic myopia (mCNV) under real-world conditions in Germany. One eye per patient was included. The OCEAN study was approved by the ethics committee of the Eberhard Karls University, Tuebingen, Germany, and the competent regulatory authority was notified. All patients provided written consent. All patients were treated with intravitreal injections of 0.5 mg ranibizumab, with treatment performed at the physicians' discretion. Data including baseline characteristics, BCVA results over time, results of OCT and FA examinations, as well as adverse events were collected between December 2011 and December 2016. The design and patient demographics have been previously described in detail [24].

### 2.2. Participants and Study Centres

Data were collected prospectively by the treating physicians. The participating OCEAN study sites are provided in the Supplementary Table S1. At baseline, demographics, clinical characteristics, previous treatments, BCVA, and the results from OCT and FA were recorded. In subsequent visits, information including the date, whether an injection was administered or not (including the reason behind the decision), BCVA and OCT measurements, and any adverse events (AEs) was recorded.

Participants were excluded from the OCEAN study if there were contraindications based on the Summary of Product Characteristics, if they had received intravitreal anti-VEGF treatment of the study eye in the three months prior to enrollment, or if they received previous intravitreal steroid treatment.

The subset of patients with nAMD was recruited by 347 (94.0%) of the 369 OCEAN study centres—85 general ophthalmology practices (of the approximately 4,700 in Germany), and 262 retina specialist centres (of the approximately 1,200 in Germany).

### 2.3. Data Analysis

In order to reflect a minimum of regularity in terms of BCVA examinations, a subsample of well-followed nAMD patients were selected for the analysis. This well-followed subsample included patients without study discontinuation until Month 24, with a maximum duration between subsequent BCVA examinations of 60 days and with a maximum mean duration between subsequent BCVA examinations of 45 days (similar to Framme et al. [15]).

To analyse the impact of the frequency of OCT examinations, three subgroups of patients within the well-followed subsample were created according to the number of OCT examinations received over the 24-month course of the study. Three subgroups were created by examining the distribution of OCT examinations and were classified as “rarely OCT examined” (those who received ≤4 OCT examinations), “moderately OCT examined” (those who received 5–8 OCT examinations), and “well-monitored by OCT” (those who received ≥8 OCT examinations with a maximum of 250 days between subsequent OCT examinations). For further analyses, the “moderately OCT examined” group was omitted so that a better selective comparison could be made to show the impact of OCT diagnostics on treatment decisions. To adjust for the potential influence of baseline BCVA between the patient subgroups, pairwise matching between “rarely OCT examined” and “well monitored by OCT” subgroups using a propensity score based on baseline BCVA was applied. Values for BCVA were converted to logarithm of minimum angle of resolution (logMAR) values and Early Treatment Diabetic Retinopathy Study (ETDRS) letters.

The statistical analyses were purely descriptive and followed a predefined analysis plan. Data are presented descriptively as median (range), mean (SD), or % of patients. Time-to-response (to first improvement of ≥15 letters from baseline) and duration-of-response (time from first improvement of ≥15 letters to first time point of losing this improvement) were analysed using Kaplan–Meier estimates.

## 3. Results

### 3.1. Study Population

Of the 5,641 OCEAN patients, 3,631 patients (64.4%) were diagnosed with nAMD. The mean age of the nAMD patients was 77.9 ± 8.1 years, and the majority of patients were female (61.2%) (Table 1).

**Figure 1:**
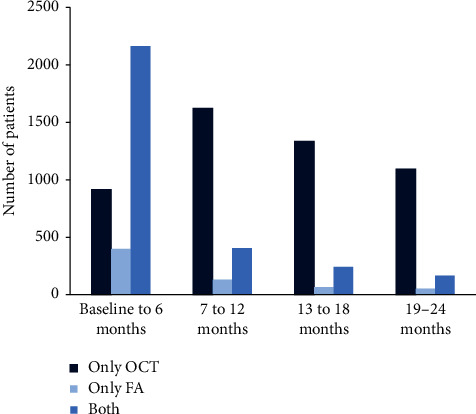
Number of patients receiving OCT, FA, and combined OCT + FA examinations per 6-month period of the OCEAN study. FA: fluorescein angiography; OCT: optical coherence tomography.

In the first 6 months of the OCEAN study, most patients (59.6%) received a combination of OCT and FA examinations of the total 3,631 examinations performed. For the remaining 7,073 examinations performed over the course of treatment, the occurrence of FA examinations in combination with OCT examinations was low (occurring in 7.6% to 13.4% of 6-month periods), with patients being more likely to receive only OCT examinations (occurring in 48.9% to 52.5% of 6-month periods) (Figure 1). The median number of OCT examinations was 3 in the first 12 months (range: 0–13; mean: 3.8) and 4 over 24 months (range: 0–25; mean: 5.9).

The well-followed subsample included 1,153 nAMD patients who were included in further analyses for this study. This subsample was divided into three subgroups based on the distribution of OCT examinations received over the 24-month course of the study: the “rarely OCT examined” subgroup included patients with ≤4 OCT examinations, the “moderately OCT examined” subgroup included patients with 5–7 OCT examinations, and the subgroup “well-monitored by OCT” subgroup included patients with ≥8 OCT with a maximum of 250 days between subsequent OCT examinations. Baseline population demographics for the full nAMD study population and the three subgroups are presented in Table 1.

**Figure 2:**
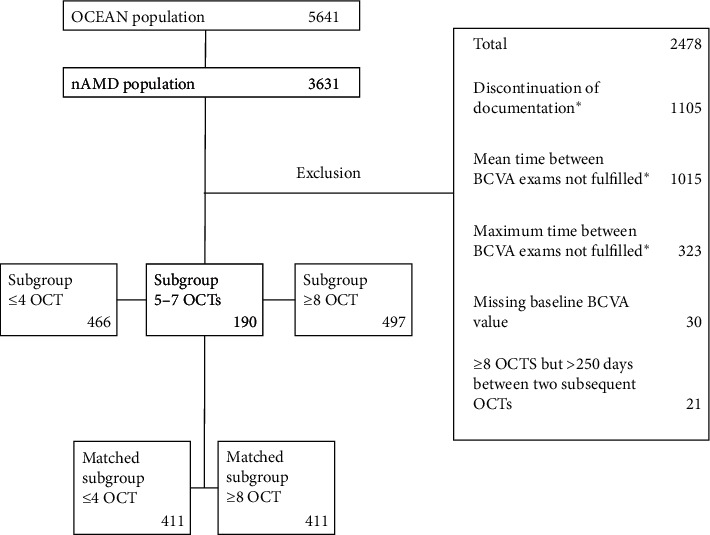
Patient disposition of participants with nAMD in the OCEAN study. ^*∗*^Multiple responses possible. BCVA: best-corrected visual acuity; nAMD: neovascular age-related macular degeneration; OCT: optical coherence tomography.

Those who received ≤4 OCT examinations (*n* = 466) and those who received ≥8 OCT examinations with a maximum of 250 days between subsequent OCT examinations (*n* = 497) were included in further analyses for selective comparison. Patients with 5–7 OCT examinations (*n* = 190) and those who received ≥8 OCT examinations but had >250 days between subsequent OCT examinations (*n* = 21) were not included in further analyses (Figure 2).

The significant difference in baseline BCVA between the ≤4 OCT examination and ≥8 OCT examination groups detected before propensity score matching was successfully eliminated through matching. Since matching was only performed using one variable, it was expected that some differences between groups would remain significant. After matching, the two groups had similar baseline characteristics, with only pretreatment status remaining significant (Table 2). The resulting subsample contained 411 patients per group.

### 3.2. Treatment and Monitoring in relation to Number of OCT Examinations

**Figure 3:**
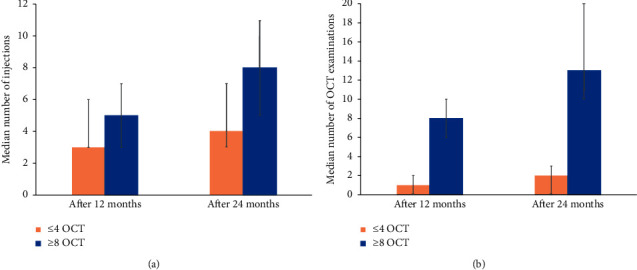
(a) Median number of ranibizumab injections with the first and third quartiles for patients receiving ≤4 OCT examinations or ≥8 OCT examinations for the matched sample (*N* = 411 for both groups); (b) median number of OCT examinations with the first and third quartiles for patients receiving ≤4 OCT examinations or ≥8 OCT examinations for the matched sample (*N* = 411 for both groups). OCT: optical coherence tomography.

For the subgroup with ≤4 OCT examinations, the median number of OCT examinations was 1 over 12 months (range: 0–4; mean: 1.3) and 2 over 24 months (range: 0–4; mean: 1.6). For the subgroup with ≥8 OCT examinations, the median number of OCT examinations was 8 over 12 months (range: 0–13; mean: 8.0) and 13 over 24 months (range: 8–25; mean: 14.7) (Figure 3). When assessing the number of ranibizumab injections after the first 12 months, patients with ≤4 OCT examinations had received a median of 3 injections (range: 1–13; mean 4.35), while those with ≥8 OCT examinations had received a median of 5 injections (range: 1–12; mean 5.42). After the full observation period of 24 months, patients with ≤4 OCT examinations had received a median of 4 injections (range: 1–19; mean 5.45), while patients with ≥8 OCT examinations had received a median of 8 injections (range: 1–21; mean 8.19) (Figure 3).

For each 6-month period of the study, patients with ≥8 OCT examinations received overall more injections than those with ≤4 OCT examinations: the median number of injections was greatest within the first 6 months (3 for patients with ≤4 OCT examinations (range: 1–8; mean: 3.47) and 4 for patients with ≥8 OCT examinations (range: 1–7; mean: 3.78)) and decreased over the remaining observation period, with the lowest median number of injections observed in the last 6 months of the study (0 for patients with ≤4 OCT examinations (range: 0–7; mean: 0.52) and 1 for patients with ≥8 OCT examinations (range: 0–5; mean: 1.22)).

For patients receiving ≤4 OCT examinations, a median of 9 BCVA examinations were performed over the first 12 months (range: 2–13; mean: 8.7) and 14 BCVA examinations over 24 months (range: 2–25; mean: 14.7). Patients receiving ≥8 OCT examinations had a median of 11 BCVA examinations after 12 months (range: 7–13; mean: 11.3) and 22 BCVA examinations after 24 months (range: 7–25; mean: 21.1). During the first 12 months, 278 patients (67.6%) with ≤4 OCT examinations and all patients with ≥8 OCT examinations had at least 7 documented BCVA examinations.

**Figure 4:**
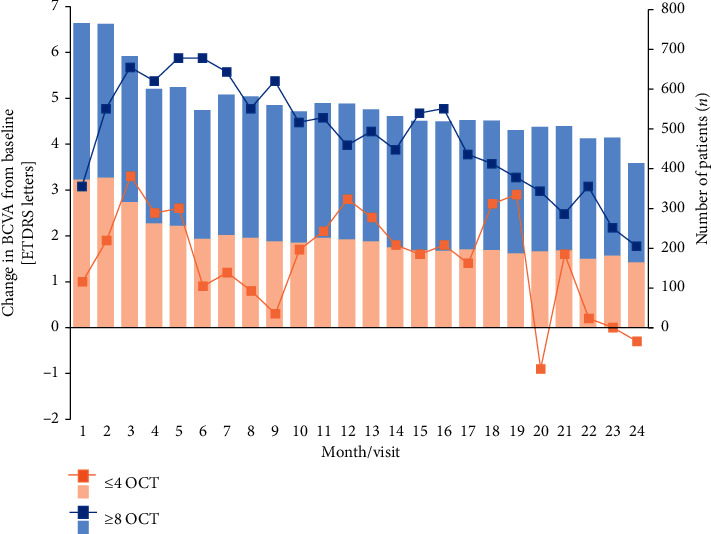
Changes in BCVA from baseline in ETDRS letters for patients receiving ≤4 OCT examinations or ≥8 OCT examinations. BCVA: best-corrected visual acuity; ETDRS: Early Treatment for Diabetic Retinopathy Study; OCT: optical coherence tomography.

Patients receiving ≤4 OCT examinations had a mean change of BCVA in ETDRS letters from baseline of +2.8 (±20.0) at 12 months (*n* = 223) and −0.3 (±26.1) at 24 months (*n* = 165), while patients receiving ≥8 OCT examinations showed changes in +4.2 (±20.0) at 12 months (*n* = 341) and +2.0 (±20.8) at 24 months (*n* = 249). The trend in change in BCVA is presented in Figure 4.

**Figure 5:**
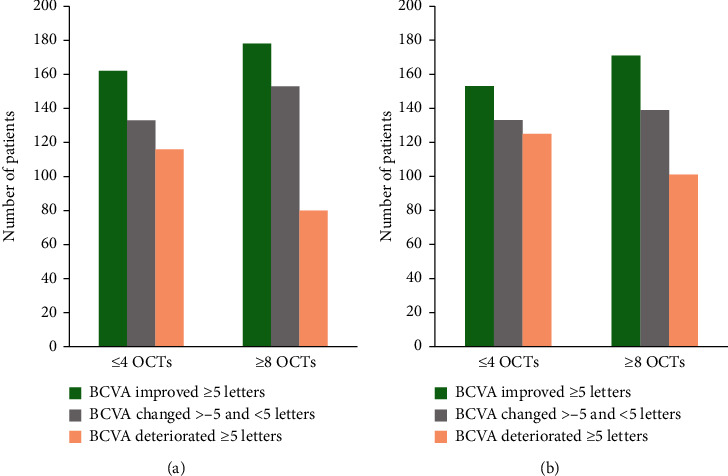
Treatment response from baseline to 12 and 24 months for patients receiving ≤4 OCT or ≥8 OCT examinations for the matched sample (*N* = 411 for both groups). BCVA: best-corrected visual acuity; OCT: optical coherence tomography. (a) 12 months; (b) 24 months.

Of the patients receiving ≤4 OCT examinations, 162 (39.4%) showed a BCVA improvement of ≥5 letters from baseline after 12 months, while such an improvement was found in 178 patients (43.3%) receiving ≥8 OCT examinations. A deterioration of BCVA of ≥5 letters from baseline after 12 months was observed in 116 patients (28.2%) receiving ≤4 OCT examinations, while 80 patients (19.5%) receiving ≥8 OCT examinations showed this deterioration. After 24 months, 153 patients (37.2%) receiving ≤4 OCT examinations and 171 patients (41.6%) receiving ≥8 OCT examinations showed a BCVA improvement of ≥5 letters from baseline. A deterioration of BCVA of ≥5 letters from baseline after 24 months was observed in 125 patients (30.4%) receiving ≤4 OCT examinations and in 101 patients (24.6%) receiving ≥8 OCT examinations (Figure 5).

**Figure 6:**
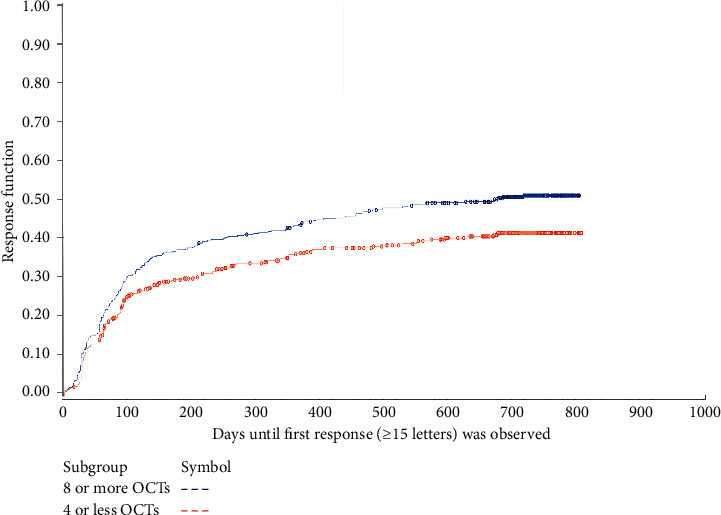
Days until the first response was observed (change from baseline of ≥15 letters) for patients receiving ≤4 OCT or ≥8 OCT examinations. OCT: optical coherence tomography.

**Figure 7:**
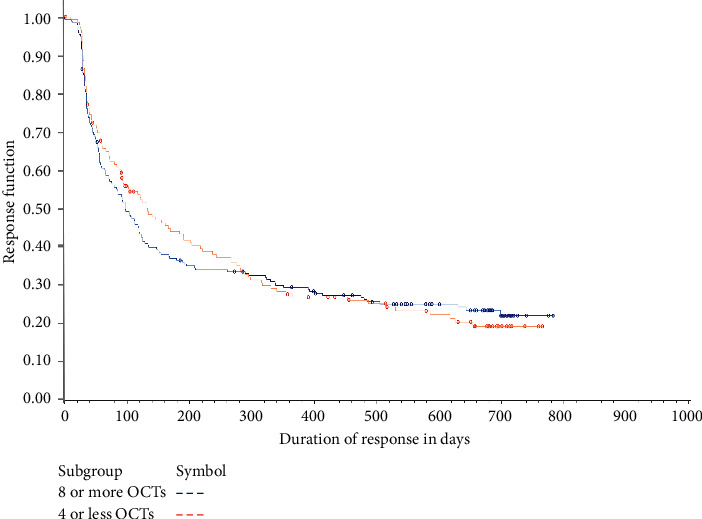
Duration-of-response of ≥15 letters for patients receiving ≤4 OCT or ≥8 OCT examinations. OCT: optical coherence tomography.

The time-to-response was greater for patients receiving ≤4 OCT examinations than for patients receiving ≥8 OCT examinations (Figure 6). About 25% of patients from both OCT groups had a ≥15 letter response within the first 3 months. Thereafter, the response rate was higher in patients receiving ≥8 OCT examinations compared to those receiving ≤4 OCT examinations. In patients who showed a ≥15 letter response, the duration-of-response was similar between the two groups. The probability of a ≥15 letter response lasting ≥3 months was 58% for patients receiving ≤4 OCT examinations and 53% for patients receiving ≥8 OCT examinations, while the probability of a ≥15 letter response lasting ≥12 months was 26% and 29%, respectively (Figure 7).

### 3.3. Regularity of OCT Examinations and Injections

**Figure 8:**
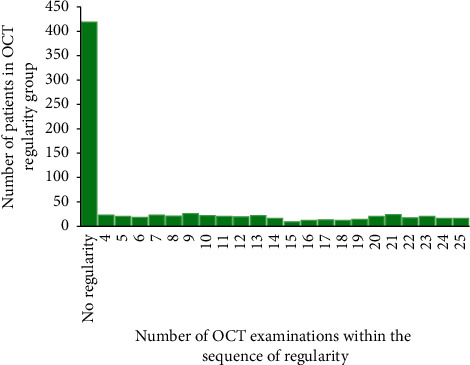
Number of OCT examinations within the sequence of regularity. Note: for OCT examinations to be considered regular in the initial loading phase during the first 3 months, subsequent OCT examinations had to occur within 21 and 60 days and the difference in any of the intervals could not be more than 30 days. For OCT examinations to be considered regular in the later maintenance phase, subsequent OCT examinations had to occur within 21 and 90 days and the difference in any of the intervals could not be more than 45 days. A sequence of regularity had to contain at least 4 OCT examinations. OCT: optical coherence tomography.

A total of 419 patients (51.0%) showed no regularity in their OCT examinations as defined below. The remaining patients were fairly equally distributed, having between 4 and 25 OCT examinations in the sequence of regularity (Figure 8). Regularity of OCT examinations was defined based on the time between subsequent OCT examinations, the time interval between OCT examinations, and the patient's treatment phase (either in the initial loading phase of the first 3 injections or in a later stage of treatment maintenance). For OCT examinations to be considered regular in the initial loading phase, subsequent OCT examinations had to occur within 21 and 60 days and the difference in any of the intervals could not be more than 30 days. For OCT examinations to be considered regular in the maintenance phase, subsequent OCT examinations had to occur within 21 and 90 days and the difference in any of the intervals could not be more than 45 days. A sequence of regularity had to contain at least 4 OCT examinations.

**Figure 9:**
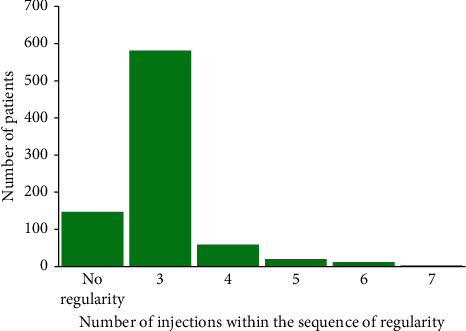
Number of injections within the sequence of regularity. Note: a sequence of injections was defined as regular if at least 3 injections occurred within the same individual time frame (±10 days) and with the sequence starting on the 6th visit at the latest.

A total of 147 patients (17.9%) showed no regularity in their injections. The majority of patients (*n* = 581, 70.7%) had 3 regular injections (Figure 9). A sequence of injections was defined as regular if at least 3 injections occurred within the same individual time frame (±10 days) and if the sequence started on the 6th visit at the latest.

## 4. Discussion

To our knowledge, the real-life observational OCEAN study is the first study demonstrating that infrequent use of OCT imaging for retreatment decisions might lead to or accompany unfavorable treatment outcomes in patients with nAMD undergoing anti-VEGF treatment. Even when looking at patients with regular injections, an infrequent use of OCT and FA was identified between 2011 and 2016 in Germany. As sufficient retreatment in accordance with the recommendations of the German ophthalmological societies and drug label is only possible either using fixed injection intervals or with as-needed treatment regimens based on morphologic activity criteria (PRN and T&E), the observed follow-up and examination strategies might explain at least parts of the observed undertreatment. In particular, this is supported by the observation that more frequent OCT examinations were associated with a higher number of treatments.

One potential reason for the low frequency of OCT imaging observed might be the costs associated with performing OCT examination in clinical practice. There was limited reimbursement for OCT imaging in Germany before October 2019. Therefore, current and future imaging strategies will likely differ from the ones observed in this study. Additionally, in real-life setting, there is the potential for nonadherence and nonpersistence which could have exacerbated the associated shorter time-to-response and a higher number of OCT examinations/visits.

The results underline the previous body of evidence that OCT is a powerful and effective tool to noninvasively assess the activity of nAMD [25]. Determining activity of the disease using OCT either influences the decision for or against treatment (PRN) or for a shortening or extension of the treatment/control intervals (T&E). Some argue for the possibility of carrying out the examination directly without pupil dilation (although this should not be done for all visits and patients) [26].

As per the study aims, both patients with and patients without OCT monitoring were included in the OCEAN study in order to draw comparisons between these groups. However, even the patients who received OCT monitoring had no OCT examinations during most study visits, limiting the comparisons that could be made [27]. This also limits the conclusions that can be drawn, as even patients with OCT examinations were examined so irregularly and rarely over time. The control of interpretations and possible misinterpretations—as seen in the CATT trial—is not even considered here yet. Although just “OCT” was written in the study protocol, the use of spectral domain (SD-OCT) had mostly replaced time domain machines (TD-OCT) during the period of the study [28]. Further evaluations must be awaited, which describe a subsequent second diagnosis of the images by several reading centres [29, 30]. Hopefully, the results will shed some light on why patients were so rarely treated despite ophthalmologist visits and OCT examinations.

After it was discovered that the follow-up inspection was inadequately handled, payers and quality assurers should control and incentivise more strictly the use of OCT. There is no alternative to implement individualized treatment regimens, even when theoretical arguments for fixed treatment regimens might exist in some patients. Arguments against possible overtreatment by a fixed treatment scheme include, for example, the cumulative growing risk of endophthalmitis. Each individualized treatment regime needs a regular assessment of active macular neovascularization activity—this is not possible in the absence of adequate retinal imaging [31]. Without knowing the activity of the disease, unnecessary treatments cannot be ruled out. But, looking more closely at the individual OCT images would allow over- and undertreatment to be contained. The data confirmed again a high amount of undertreatment in Germany [32–34]. Like in many other countries, tight monitoring and consistent retreatment are important basic requirements for successful treatment.

### 4.1. Limitations

Important limitations must be considered in the light of the study. Although the noninterventional character should not influence the daily practice of doctors, observational artefacts and selection over time might contribute to the potential amount of bias. Only a subset of 1,153 patients was reported here to achieve a minimum density of observation points, which probably led to an even more pronounced selection bias. The selection might even superimpose the association of more frequent diagnostics and the stricter follow-up. Therefore, no causal relationship between the use of OCT and the individual treatment decision can be made. Since a difference of visual became apparent after the loading dose, the use of the OCT examination—unconsciously or planned—could also depend on this first response. Facing the large heterogeneity, the study was not designed to evaluate the individualized treatment approach. Especially in the group with rare OCT assessments, this has a strong effect in the form of a strong fluctuation of mean visual acuity. Last but not least, the high rate of loss of follow-up increases the uncertainty and limits the statistical representativeness.

## 5. Conclusions

The results indicate that the determination of active macular neovascularization activity by OCT was used less frequently in Germany between 2011 and 2016 for follow-up and retreatment decisions than in prospective AMD clinical trials [35]. Patients with earlier response and more favourable development of visual function in particular received more regular OCT examinations.

In addition to nonadherence and concomitant diseases, the low number of visits as well as high number of treatment decisions made without the use of OCT may lead to undertreatment and poorer functional outcomes in patients undergoing ranibizumab treatment for nAMD in Germany. One potential reason for this could be that OCT was not covered by insurance for all patients.

Furthermore, this study identified that physicians may not always strictly follow the medical guidelines, which could contribute to the suspected undertreatment and thus to worse results compared to RCTs [36]. OCT is an effective tool, and its correct use should be promoted through trainings and updates directed at physicians [37]. Furthermore, measuring actual practice behaviours of retina specialists, particularly as they relate to established retreatment schemes, is challenging, but essential.

## Figures and Tables

**Table 1:** Patient demographics and baseline disease characteristics of OCEAN nAMD population and the three OCT examinations subgroups.

	Total nAMD population, *N* = 3631	≤4 OCT subgroup, *N* = 466	5–7 OCT subgroup, *N* = 190	≥8 OCT subgroup, *N* = 497
Gender (*n* (%))				
Male	1401 (38.6%)	193 (41.4%)	76 (40.0%)	191 (38.4%)
Female	2222 (61.2%)	273 (58.6%)	114 (60.0%)	306 (61.6%)
Missing	8 (0.2%)	0	0	0

Age (years)				
*n*	3623	466	190	497
Mean (SD)	77.9 (8.1)	77.9 (8.9)	76.9 (8.9)	77.3 (8.2)

Pretreatment status^*∗*^				
Treatment-naïve	2657 (73.2%)	370 (79.4%)	134 (70.5%)	364 (73.2%)
Pretreated with anti-VEGF	631 (17.4%)	62 (13.3%)	32 (16.8%)	109 (21.9%)
Possibly pretreated	343 (9.5%)	34 (7.3%)	24 (12.6%)	24 (4.8%)

Treatment delay (days)				
*n*	3481	436	182	487
Mean (SD)	20.3 (19.4)	12.8 (13.0)	13.1 (11.3)	11.9 (10.5)
Median	15	10	13	10
Min–Max	0–90	0–79	0–47	0–57

BCVA of study eye at baseline				
*n*	3601	466	190	497
Mean (SD) ETDRS letters	52.0 (21.2)	48.3 (25.2)	53.2 (21.1)	55.1 (17.9)

Number of injections				
*n*	3631	466	190	497
Mean (SD)	5.7 (3.7)	3.0 (3.6)	7.1 (4.7)	8.4 (4.4)
Median	5	4	6	8
Min–Max	1–24	1–19	1–22	1–24

^*∗*^Pretreatment status was categorized as treatment-naïve (no previous intravitreal anti-VEGF documented and ≤90 days between diagnosis and first injection within OCEAN), pretreated (received anti-VEGF more than 3 months before study entry), and possibly pretreated (all remaining patients not meeting the other criteria). BCVA: best-corrected visual acuity; ETDRS: Early Treatment for Diabetic Retinopathy Study; Max: maximum; Min: minimum; nAMD: neovascular age-related macular degeneration; OCT: optical coherence tomography; SD: standard deviation; VEGF: vascular endothelial growth factor.

**Table 2:** Patient demographics and baseline disease characteristics of the matched OCT examination subgroups.

	≤4 OCT matched subgroup, *N* = 411	≥8 OCT matched subgroup, *N* = 411	*p* value
Gender (*n* (%))			0.3197
Male	173 (42.1%)	159 (38.7%)	
Female	238 (57.9%)	252 (61.3%)	
Missing	0	0	

Age (years)			0.8678
*n*	411	411	
Mean (SD)	77.5 (9.0)	77.4 (8.2)	

Pretreatment status^*∗*^			0.0003
Treatment-naïve	331 (80.5%)	300 (73.0%)	
Pretreated with anti-VEGF	51 (12.4%)	93 (22.6%)	
Possibly pretreated	29 (7.1%)	18 (4.4%)	

Treatment delay (days)			0.2940
*n*	385	402	
Mean (SD)	12.8 (13.0)	11.9 (10.9)	
Median	10	9	
Min–Max	0–79	0–57	

BCVA of study eye at baseline			1.0000
*n*	411	411	
Mean (SD) ETDRS letters	53.7 (18.4)	53.7 (18.4)	

^*∗*^Pretreatment status was categorized as treatment-naïve (no previous intravitreal anti-VEGF documented and ≤90 days between diagnosis and first injection within OCEAN), pretreated (received anti-VEGF more than 3 months before study entry), and possibly pretreated (all patients not meeting the treatment-naïve or pretreated criteria). BCVA: best-corrected visual acuity; ETDRS: Early Treatment for Diabetic Retinopathy Study; Max: maximum; Min: minimum; nAMD: neovascular age-related macular degeneration; OCT: optical coherence tomography; SD: standard deviation; VEGF: vascular endothelial growth factor.

## Data Availability

The datasets generated during and/or analysed during the current study are not publicly available due to German data protection law but are available from the corresponding author on reasonable request. The regulatory requirements allow anonymous data records to be viewed if requested by third parties.

## Supplementary Materials

Supplementary Materials Supplementary Table 1. Patient-based adverse events—MedDRA primary SOCs—and preferred terms (multiple responses)—SES.
