# Supplementary material for: Use of Imaging Modalities in Real Life: Impact on Visual Acuity Outcomes of Ranibizumab Treatment for Neovascular Age-Related Macular Degeneration in Germany
Source: J Ophthalmol. 2020 Jul 16;2020:8024258. doi: 10.1155/2020/8024258 (PMC7382751; doi:10.1155/2020/8024258)
Supplement: Supplementary Materials — Supplementary Table 1. Patient-based adverse events—MedDRA primary SOCs—and preferred terms (multiple responses)—SES. [file 8024258.f1.docx]

**Supplementary Table 1.** Patient-based Adverse Events – MedDRA primary SOCs and Preferred Terms (multiple responses) - SES

|  |  | Number of patients | Number of patients | Number of patients | Number of patients | Number of patients |
| --- | --- | --- | --- | --- | --- | --- |
|  |  | Total | Total | Total | Total | Total |
|  |  | N (%) | N (%) | N (%) | N (%) | N (%) |
| Patients SAF |  | 5781 ( 100.0%) | 451 ( 100.0%) | 417 ( 100.0%) | 498 ( 100.0%) | 213 ( 100.0%) |
|  |  |  |  |  |  |  |
| Eye disorders | All Patients | 543 ( 9.4%) | 193 ( 42.8%) | 148 ( 35.5%) | 204 ( 41.0%) | 105 ( 49.3%) |
|  | Visual acuity reduced | 201 ( 3.5%) | 34 ( 7.5%) | 49 ( 11.8%) | 82 ( 16.5%) | 39 ( 18.3%) |
|  | Retinal haemorrhage | 43 ( 0.7%) | 7 ( 1.6%) | 18 ( 4.3%) | 9 ( 1.8%) | 9 ( 4.2%) |
|  | Posterior capsule opacification | 42 ( 0.7%) | 27 ( 6.0%) | 5 ( 1.2%) | 10 ( 2.0%) | 1 ( 0.5%) |
|  | Cataract | 38 ( 0.7%) | 24 ( 5.3%) | 9 ( 2.2%) | 2 ( 0.4%) | 3 ( 1.4%) |
|  | Vitreous haemorrhage | 31 ( 0.5%) | 4 ( 0.9%) | 19 ( 4.6%) | 2 ( 0.4%) | 6 ( 2.8%) |
|  | Macular oedema | 30 ( 0.5%) | 13 ( 2.9%) | 6 ( 1.4%) | 8 ( 1.6%) | 3 ( 1.4%) |
|  | Retinal oedema | 26 ( 0.4%) | 5 ( 1.1%) | 2 ( 0.5%) | 19 ( 3.8%) | 0 ( 0.00%) |
|  | Eye irritation | 21 ( 0.4%) | 6 ( 1.3%) | 0 ( 0.00%) | 15 ( 3.0%) | 0 ( 0.00%) |
|  | Glaucoma | 19 ( 0.3%) | 4 ( 0.9%) | 8 ( 1.9%) | 1 ( 0.2%) | 6 ( 2.8%) |
|  | Retinal cyst | 18 ( 0.3%) | 5 ( 1.1%) | 1 ( 0.2%) | 10 ( 2.0%) | 2 ( 0.9%) |
|  | Ocular hypertension | 17 ( 0.3%) | 9 ( 2.0%) | 3 ( 0.7%) | 3 ( 0.6%) | 3 ( 1.4%) |
|  | Retinal disorder | 17 ( 0.3%) | 3 ( 0.7%) | 0 ( 0.00%) | 13 ( 2.6%) | 1 ( 0.5%) |
|  | Subretinal fluid | 16 ( 0.3%) | 3 ( 0.7%) | 1 ( 0.2%) | 8 ( 1.6%) | 4 ( 1.9%) |
|  | Neovascular age-related macular degeneration | 15 ( 0.3%) | 3 ( 0.7%) | 5 ( 1.2%) | 4 ( 0.8%) | 4 ( 1.9%) |
|  | Detachment of retinal pigment epithelium | 13 ( 0.2%) | 0 ( 0.00%) | 5 ( 1.2%) | 1 ( 0.2%) | 7 ( 3.3%) |
|  | Macular scar | 13 ( 0.2%) | 1 ( 0.2%) | 7 ( 1.7%) | 1 ( 0.2%) | 4 ( 1.9%) |
|  | Retinal vein occlusion | 13 ( 0.2%) | 1 ( 0.2%) | 6 ( 1.4%) | 0 ( 0.00%) | 6 ( 2.8%) |
|  | Retinal degeneration | 12 ( 0.2%) | 1 ( 0.2%) | 4 ( 1.0%) | 1 ( 0.2%) | 6 ( 2.8%) |
|  | Eye pain | 11 ( 0.2%) | 2 ( 0.4%) | 1 ( 0.2%) | 5 ( 1.0%) | 3 ( 1.4%) |
|  | Metamorphopsia | 11 ( 0.2%) | 0 ( 0.00%) | 1 ( 0.2%) | 10 ( 2.0%) | 0 ( 0.00%) |
|  | Retinal scar | 11 ( 0.2%) | 0 ( 0.00%) | 4 ( 1.0%) | 4 ( 0.8%) | 3 ( 1.4%) |
|  | Corneal erosion | 10 ( 0.2%) | 3 ( 0.7%) | 0 ( 0.00%) | 6 ( 1.2%) | 1 ( 0.5%) |
|  | Retinal ischaemia | 10 ( 0.2%) | 1 ( 0.2%) | 4 ( 1.0%) | 2 ( 0.4%) | 3 ( 1.4%) |
|  | Choroidal neovascularisation | 9 ( 0.2%) | 3 ( 0.7%) | 3 ( 0.7%) | 2 ( 0.4%) | 1 ( 0.5%) |
|  | Conjunctival haemorrhage | 9 ( 0.2%) | 3 ( 0.7%) | 0 ( 0.00%) | 5 ( 1.0%) | 1 ( 0.5%) |
|  | Ocular hyperaemia | 9 ( 0.2%) | 5 ( 1.1%) | 0 ( 0.00%) | 2 ( 0.4%) | 2 ( 0.9%) |
|  | Retinal detachment | 9 ( 0.2%) | 1 ( 0.2%) | 5 ( 1.2%) | 0 ( 0.00%) | 3 ( 1.4%) |
|  | Retinal pigment epithelial tear | 9 ( 0.2%) | 0 ( 0.00%) | 4 ( 1.0%) | 0 ( 0.00%) | 5 ( 2.3%) |
|  | Diabetic retinopathy | 8 ( 0.1%) | 3 ( 0.7%) | 3 ( 0.7%) | 1 ( 0.2%) | 1 ( 0.5%) |
|  | Vision blurred | 8 ( 0.1%) | 1 ( 0.2%) | 1 ( 0.2%) | 4 ( 0.8%) | 2 ( 0.9%) |
|  | Conjunctival irritation | 7 ( 0.1%) | 1 ( 0.2%) | 0 ( 0.00%) | 6 ( 1.2%) | 0 ( 0.00%) |
|  | Visual impairment | 7 ( 0.1%) | 2 ( 0.4%) | 0 ( 0.00%) | 5 ( 1.0%) | 0 ( 0.00%) |
|  | Open angle glaucoma | 6 ( 0.1%) | 1 ( 0.2%) | 2 ( 0.5%) | 2 ( 0.4%) | 1 ( 0.5%) |
|  | Subretinal fibrosis | 6 ( 0.1%) | 0 ( 0.00%) | 3 ( 0.7%) | 0 ( 0.00%) | 3 ( 1.4%) |
|  | Age-related macular degeneration | 5 ( 0.1%) | 0 ( 0.00%) | 1 ( 0.2%) | 1 ( 0.2%) | 3 ( 1.4%) |
|  | Blindness | 5 ( 0.1%) | 0 ( 0.00%) | 1 ( 0.2%) | 0 ( 0.00%) | 4 ( 1.9%) |
|  | Dry eye | 5 ( 0.1%) | 4 ( 0.9%) | 0 ( 0.00%) | 1 ( 0.2%) | 0 ( 0.00%) |
|  | Lacrimation increased | 5 ( 0.1%) | 3 ( 0.7%) | 0 ( 0.00%) | 2 ( 0.4%) | 0 ( 0.00%) |
|  | Macular fibrosis | 5 ( 0.1%) | 2 ( 0.4%) | 2 ( 0.5%) | 0 ( 0.00%) | 1 ( 0.5%) |
|  | Retinopathy proliferative | 5 ( 0.1%) | 1 ( 0.2%) | 2 ( 0.5%) | 1 ( 0.2%) | 1 ( 0.5%) |
|  | Blepharitis | 4 ( 0.1%) | 4 ( 0.9%) | 0 ( 0.00%) | 0 ( 0.00%) | 0 ( 0.00%) |
|  | Cystoid macular oedema | 4 ( 0.1%) | 2 ( 0.4%) | 0 ( 0.00%) | 1 ( 0.2%) | 1 ( 0.5%) |
|  | Eye disorder | 4 ( 0.1%) | 2 ( 0.4%) | 2 ( 0.5%) | 1 ( 0.2%) | 0 ( 0.00%) |
|  | Macular degeneration | 4 ( 0.1%) | 1 ( 0.2%) | 2 ( 0.5%) | 1 ( 0.2%) | 0 ( 0.00%) |
|  | Retinal aneurysm | 4 ( 0.1%) | 1 ( 0.2%) | 1 ( 0.2%) | 1 ( 0.2%) | 1 ( 0.5%) |
|  | Retinal exudates | 4 ( 0.1%) | 3 ( 0.7%) | 0 ( 0.00%) | 1 ( 0.2%) | 0 ( 0.00%) |
|  | Retinal vein thrombosis | 4 ( 0.1%) | 1 ( 0.2%) | 3 ( 0.7%) | 0 ( 0.00%) | 0 ( 0.00%) |
|  | Vitreous adhesions | 4 ( 0.1%) | 2 ( 0.4%) | 1 ( 0.2%) | 1 ( 0.2%) | 0 ( 0.00%) |
|  | Eye haemorrhage | 3 ( 0.1%) | 0 ( 0.00%) | 2 ( 0.5%) | 0 ( 0.00%) | 1 ( 0.5%) |
|  | Macular cyst | 3 ( 0.1%) | 1 ( 0.2%) | 2 ( 0.5%) | 0 ( 0.00%) | 0 ( 0.00%) |
|  | Macular hole | 3 ( 0.1%) | 0 ( 0.00%) | 2 ( 0.5%) | 0 ( 0.00%) | 1 ( 0.5%) |
|  | Maculopathy | 3 ( 0.1%) | 1 ( 0.2%) | 1 ( 0.2%) | 1 ( 0.2%) | 0 ( 0.00%) |
|  | Ocular discomfort | 3 ( 0.1%) | 2 ( 0.4%) | 0 ( 0.00%) | 1 ( 0.2%) | 0 ( 0.00%) |
|  | Punctate keratitis | 3 ( 0.1%) | 2 ( 0.4%) | 0 ( 0.00%) | 1 ( 0.2%) | 0 ( 0.00%) |
|  | Vitreous detachment | 3 ( 0.1%) | 0 ( 0.00%) | 1 ( 0.2%) | 1 ( 0.2%) | 1 ( 0.5%) |
|  | Vitreous disorder | 3 ( 0.1%) | 1 ( 0.2%) | 0 ( 0.00%) | 2 ( 0.4%) | 0 ( 0.00%) |
|  | Vitreous floaters | 3 ( 0.1%) | 0 ( 0.00%) | 0 ( 0.00%) | 3 ( 0.6%) | 0 ( 0.00%) |
|  | Abnormal sensation in eye | 2 ( 0.0%) | 2 ( 0.4%) | 0 ( 0.00%) | 0 ( 0.00%) | 0 ( 0.00%) |
|  | Anterior chamber cell | 2 ( 0.0%) | 0 ( 0.00%) | 0 ( 0.00%) | 1 ( 0.2%) | 1 ( 0.5%) |
|  | Anterior chamber disorder | 2 ( 0.0%) | 0 ( 0.00%) | 0 ( 0.00%) | 2 ( 0.4%) | 0 ( 0.00%) |
|  | Conjunctival oedema | 2 ( 0.0%) | 1 ( 0.2%) | 0 ( 0.00%) | 1 ( 0.2%) | 0 ( 0.00%) |
|  | Conjunctivitis allergic | 2 ( 0.0%) | 1 ( 0.2%) | 0 ( 0.00%) | 1 ( 0.2%) | 0 ( 0.00%) |
|  | Diabetic retinal oedema | 2 ( 0.0%) | 0 ( 0.00%) | 1 ( 0.2%) | 0 ( 0.00%) | 1 ( 0.5%) |
|  | Diplopia | 2 ( 0.0%) | 0 ( 0.00%) | 2 ( 0.5%) | 0 ( 0.00%) | 0 ( 0.00%) |
|  | Eye allergy | 2 ( 0.0%) | 2 ( 0.4%) | 0 ( 0.00%) | 0 ( 0.00%) | 0 ( 0.00%) |
|  | Eye pruritus | 2 ( 0.0%) | 2 ( 0.4%) | 0 ( 0.00%) | 0 ( 0.00%) | 0 ( 0.00%) |
|  | Eyelid oedema | 2 ( 0.0%) | 0 ( 0.00%) | 0 ( 0.00%) | 2 ( 0.4%) | 0 ( 0.00%) |
|  | Foreign body sensation in eyes | 2 ( 0.0%) | 1 ( 0.2%) | 0 ( 0.00%) | 1 ( 0.2%) | 0 ( 0.00%) |
|  | Iritis | 2 ( 0.0%) | 2 ( 0.4%) | 0 ( 0.00%) | 0 ( 0.00%) | 0 ( 0.00%) |
|  | Macular ischaemia | 2 ( 0.0%) | 0 ( 0.00%) | 1 ( 0.2%) | 0 ( 0.00%) | 1 ( 0.5%) |
|  | Ophthalmoplegia | 2 ( 0.0%) | 0 ( 0.00%) | 1 ( 0.2%) | 0 ( 0.00%) | 1 ( 0.5%) |
|  | Optic atrophy | 2 ( 0.0%) | 0 ( 0.00%) | 1 ( 0.2%) | 0 ( 0.00%) | 1 ( 0.5%) |
|  | Optic disc disorder | 2 ( 0.0%) | 0 ( 0.00%) | 2 ( 0.5%) | 0 ( 0.00%) | 0 ( 0.00%) |
|  | Photopsia | 2 ( 0.0%) | 1 ( 0.2%) | 0 ( 0.00%) | 1 ( 0.2%) | 0 ( 0.00%) |
|  | Retinal artery occlusion | 2 ( 0.0%) | 0 ( 0.00%) | 0 ( 0.00%) | 0 ( 0.00%) | 2 ( 0.9%) |
|  | Retinal depigmentation | 2 ( 0.0%) | 0 ( 0.00%) | 2 ( 0.5%) | 0 ( 0.00%) | 0 ( 0.00%) |
|  | Retinal fibrosis | 2 ( 0.0%) | 0 ( 0.00%) | 0 ( 0.00%) | 1 ( 0.2%) | 1 ( 0.5%) |
|  | Retinal neovascularisation | 2 ( 0.0%) | 0 ( 0.00%) | 2 ( 0.5%) | 0 ( 0.00%) | 0 ( 0.00%) |
|  | Vitritis | 2 ( 0.0%) | 1 ( 0.2%) | 0 ( 0.00%) | 0 ( 0.00%) | 1 ( 0.5%) |
|  | Amaurosis | 1 ( 0.0%) | 0 ( 0.00%) | 0 ( 0.00%) | 0 ( 0.00%) | 1 ( 0.5%) |
|  | Amaurosis fugax | 1 ( 0.0%) | 0 ( 0.00%) | 0 ( 0.00%) | 0 ( 0.00%) | 1 ( 0.5%) |
|  | Angle closure glaucoma | 1 ( 0.0%) | 0 ( 0.00%) | 1 ( 0.2%) | 0 ( 0.00%) | 0 ( 0.00%) |
|  | Anterior capsule contraction | 1 ( 0.0%) | 1 ( 0.2%) | 0 ( 0.00%) | 0 ( 0.00%) | 0 ( 0.00%) |
|  | Blindness unilateral | 1 ( 0.0%) | 0 ( 0.00%) | 0 ( 0.00%) | 0 ( 0.00%) | 1 ( 0.5%) |
|  | Chalazion | 1 ( 0.0%) | 1 ( 0.2%) | 0 ( 0.00%) | 0 ( 0.00%) | 0 ( 0.00%) |
|  | Choroidal detachment | 1 ( 0.0%) | 0 ( 0.00%) | 1 ( 0.2%) | 0 ( 0.00%) | 0 ( 0.00%) |
|  | Choroidal haemorrhage | 1 ( 0.0%) | 1 ( 0.2%) | 0 ( 0.00%) | 0 ( 0.00%) | 0 ( 0.00%) |
|  | Conjunctival erosion | 1 ( 0.0%) | 1 ( 0.2%) | 0 ( 0.00%) | 0 ( 0.00%) | 0 ( 0.00%) |
|  | Conjunctival hyperaemia | 1 ( 0.0%) | 1 ( 0.2%) | 0 ( 0.00%) | 0 ( 0.00%) | 0 ( 0.00%) |
|  | Corneal decompensation | 1 ( 0.0%) | 0 ( 0.00%) | 0 ( 0.00%) | 0 ( 0.00%) | 1 ( 0.5%) |
|  | Corneal oedema | 1 ( 0.0%) | 1 ( 0.2%) | 0 ( 0.00%) | 0 ( 0.00%) | 0 ( 0.00%) |
|  | Dry age-related macular degeneration | 1 ( 0.0%) | 0 ( 0.00%) | 1 ( 0.2%) | 0 ( 0.00%) | 0 ( 0.00%) |
|  | Ectropion | 1 ( 0.0%) | 1 ( 0.2%) | 0 ( 0.00%) | 0 ( 0.00%) | 0 ( 0.00%) |
|  | Entropion | 1 ( 0.0%) | 1 ( 0.2%) | 0 ( 0.00%) | 0 ( 0.00%) | 0 ( 0.00%) |
|  | Episcleritis | 1 ( 0.0%) | 1 ( 0.2%) | 0 ( 0.00%) | 0 ( 0.00%) | 0 ( 0.00%) |
|  | Erythema of eyelid | 1 ( 0.0%) | 1 ( 0.2%) | 0 ( 0.00%) | 0 ( 0.00%) | 0 ( 0.00%) |
|  | Eye oedema | 1 ( 0.0%) | 0 ( 0.00%) | 0 ( 0.00%) | 1 ( 0.2%) | 0 ( 0.00%) |
|  | Eyelid haematoma | 1 ( 0.0%) | 1 ( 0.2%) | 0 ( 0.00%) | 0 ( 0.00%) | 0 ( 0.00%) |
|  | Eyelid margin crusting | 1 ( 0.0%) | 1 ( 0.2%) | 0 ( 0.00%) | 0 ( 0.00%) | 0 ( 0.00%) |
|  | Eyelid pain | 1 ( 0.0%) | 1 ( 0.2%) | 0 ( 0.00%) | 0 ( 0.00%) | 0 ( 0.00%) |
|  | Eyelid thickening | 1 ( 0.0%) | 1 ( 0.2%) | 0 ( 0.00%) | 0 ( 0.00%) | 0 ( 0.00%) |
|  | Hyphaema | 1 ( 0.0%) | 0 ( 0.00%) | 1 ( 0.2%) | 0 ( 0.00%) | 0 ( 0.00%) |
|  | Hypotony of eye | 1 ( 0.0%) | 0 ( 0.00%) | 1 ( 0.2%) | 0 ( 0.00%) | 0 ( 0.00%) |
|  | Iris haemorrhage | 1 ( 0.0%) | 0 ( 0.00%) | 0 ( 0.00%) | 0 ( 0.00%) | 1 ( 0.5%) |
|  | Iris neovascularisation | 1 ( 0.0%) | 0 ( 0.00%) | 1 ( 0.2%) | 0 ( 0.00%) | 0 ( 0.00%) |
|  | Lagophthalmos | 1 ( 0.0%) | 0 ( 0.00%) | 0 ( 0.00%) | 1 ( 0.2%) | 0 ( 0.00%) |
|  | Lens disorder | 1 ( 0.0%) | 0 ( 0.00%) | 0 ( 0.00%) | 0 ( 0.00%) | 1 ( 0.5%) |
|  | Lenticular opacities | 1 ( 0.0%) | 0 ( 0.00%) | 0 ( 0.00%) | 0 ( 0.00%) | 1 ( 0.5%) |
|  | Narrow anterior chamber angle | 1 ( 0.0%) | 0 ( 0.00%) | 0 ( 0.00%) | 0 ( 0.00%) | 1 ( 0.5%) |
|  | Non-infectious endophthalmitis | 1 ( 0.0%) | 0 ( 0.00%) | 0 ( 0.00%) | 0 ( 0.00%) | 1 ( 0.5%) |
|  | Normal tension glaucoma | 1 ( 0.0%) | 0 ( 0.00%) | 1 ( 0.2%) | 0 ( 0.00%) | 0 ( 0.00%) |
|  | Ocular ischaemic syndrome | 1 ( 0.0%) | 0 ( 0.00%) | 1 ( 0.2%) | 0 ( 0.00%) | 0 ( 0.00%) |
|  | Optic nerve cupping | 1 ( 0.0%) | 0 ( 0.00%) | 1 ( 0.2%) | 0 ( 0.00%) | 0 ( 0.00%) |
|  | Retinal pigment epitheliopathy | 1 ( 0.0%) | 1 ( 0.2%) | 0 ( 0.00%) | 0 ( 0.00%) | 0 ( 0.00%) |
|  | Retinal pigmentation | 1 ( 0.0%) | 0 ( 0.00%) | 1 ( 0.2%) | 0 ( 0.00%) | 0 ( 0.00%) |
|  | Retinal tear | 1 ( 0.0%) | 0 ( 0.00%) | 1 ( 0.2%) | 0 ( 0.00%) | 0 ( 0.00%) |
|  | Retinal telangiectasia | 1 ( 0.0%) | 0 ( 0.00%) | 1 ( 0.2%) | 0 ( 0.00%) | 0 ( 0.00%) |
|  | Retinal thickening | 1 ( 0.0%) | 0 ( 0.00%) | 1 ( 0.2%) | 0 ( 0.00%) | 0 ( 0.00%) |
|  | Retinal vasculitis | 1 ( 0.0%) | 1 ( 0.2%) | 0 ( 0.00%) | 0 ( 0.00%) | 0 ( 0.00%) |
|  | Strabismus | 1 ( 0.0%) | 0 ( 0.00%) | 0 ( 0.00%) | 0 ( 0.00%) | 1 ( 0.5%) |
|  | Trichiasis | 1 ( 0.0%) | 1 ( 0.2%) | 0 ( 0.00%) | 0 ( 0.00%) | 0 ( 0.00%) |
|  | Ulcerative keratitis | 1 ( 0.0%) | 0 ( 0.00%) | 0 ( 0.00%) | 1 ( 0.2%) | 0 ( 0.00%) |
|  | Uveitis | 1 ( 0.0%) | 0 ( 0.00%) | 1 ( 0.2%) | 0 ( 0.00%) | 0 ( 0.00%) |
|  |  |  |  |  |  |  |
| General disorders and administration site conditions | All Patients | 336 ( 5.8%) | 52 ( 11.5%) | 63 ( 15.1%) | 192 ( 38.6%) | 43 ( 20.2%) |
|  | Drug ineffective | 119 ( 2.1%) | 13 ( 2.9%) | 4 ( 1.0%) | 101 ( 20.3%) | 2 ( 0.9%) |
|  | Death | 43 ( 0.7%) | 0 ( 0.00%) | 19 ( 4.6%) | 0 ( 0.00%) | 24 ( 11.3%) |
|  | Adverse event | 31 ( 0.5%) | 4 ( 0.9%) | 0 ( 0.00%) | 26 ( 5.2%) | 1 ( 0.5%) |
|  | General physical health deterioration | 21 ( 0.4%) | 4 ( 0.9%) | 6 ( 1.4%) | 10 ( 2.0%) | 1 ( 0.5%) |
|  | Malaise | 17 ( 0.3%) | 3 ( 0.7%) | 1 ( 0.2%) | 13 ( 2.6%) | 0 ( 0.00%) |
|  | Condition aggravated | 15 ( 0.3%) | 6 ( 1.3%) | 3 ( 0.7%) | 4 ( 0.8%) | 2 ( 0.9%) |
|  | Therapy non-responder | 13 ( 0.2%) | 2 ( 0.4%) | 2 ( 0.5%) | 9 ( 1.8%) | 0 ( 0.00%) |
|  | Ill-defined disorder | 10 ( 0.2%) | 2 ( 0.4%) | 0 ( 0.00%) | 8 ( 1.6%) | 0 ( 0.00%) |
|  | Gait disturbance | 7 ( 0.1%) | 2 ( 0.4%) | 3 ( 0.7%) | 1 ( 0.2%) | 1 ( 0.5%) |
|  | Asthenia | 6 ( 0.1%) | 1 ( 0.2%) | 2 ( 0.5%) | 2 ( 0.4%) | 1 ( 0.5%) |
|  | Chest discomfort | 6 ( 0.1%) | 0 ( 0.00%) | 2 ( 0.5%) | 1 ( 0.2%) | 3 ( 1.4%) |
|  | Disease recurrence | 6 ( 0.1%) | 1 ( 0.2%) | 2 ( 0.5%) | 1 ( 0.2%) | 2 ( 0.9%) |
|  | Drug intolerance | 6 ( 0.1%) | 3 ( 0.7%) | 0 ( 0.00%) | 3 ( 0.6%) | 0 ( 0.00%) |
|  | Pain | 6 ( 0.1%) | 0 ( 0.00%) | 5 ( 1.2%) | 1 ( 0.2%) | 0 ( 0.00%) |
|  | Chest pain | 5 ( 0.1%) | 0 ( 0.00%) | 3 ( 0.7%) | 0 ( 0.00%) | 2 ( 0.9%) |
|  | Tachyphylaxis | 5 ( 0.1%) | 0 ( 0.00%) | 0 ( 0.00%) | 5 ( 1.0%) | 0 ( 0.00%) |
|  | Injection site pain | 4 ( 0.1%) | 3 ( 0.7%) | 0 ( 0.00%) | 1 ( 0.2%) | 0 ( 0.00%) |
|  | Oedema | 4 ( 0.1%) | 1 ( 0.2%) | 2 ( 0.5%) | 1 ( 0.2%) | 0 ( 0.00%) |
|  | Concomitant disease aggravated | 3 ( 0.1%) | 0 ( 0.00%) | 0 ( 0.00%) | 2 ( 0.4%) | 1 ( 0.5%) |
|  | Disease progression | 3 ( 0.1%) | 0 ( 0.00%) | 1 ( 0.2%) | 0 ( 0.00%) | 2 ( 0.9%) |
|  | Fatigue | 3 ( 0.1%) | 0 ( 0.00%) | 1 ( 0.2%) | 0 ( 0.00%) | 2 ( 0.9%) |
|  | Multiple organ dysfunction syndrome | 3 ( 0.1%) | 0 ( 0.00%) | 3 ( 0.7%) | 0 ( 0.00%) | 0 ( 0.00%) |
|  | Oedema peripheral | 3 ( 0.1%) | 0 ( 0.00%) | 3 ( 0.7%) | 0 ( 0.00%) | 0 ( 0.00%) |
|  | Therapeutic response decreased | 3 ( 0.1%) | 0 ( 0.00%) | 1 ( 0.2%) | 2 ( 0.4%) | 0 ( 0.00%) |
|  | Peripheral swelling | 2 ( 0.0%) | 2 ( 0.4%) | 0 ( 0.00%) | 0 ( 0.00%) | 0 ( 0.00%) |
|  | Therapeutic response delayed | 2 ( 0.0%) | 1 ( 0.2%) | 0 ( 0.00%) | 1 ( 0.2%) | 0 ( 0.00%) |
|  | Application site haemorrhage | 1 ( 0.0%) | 1 ( 0.2%) | 0 ( 0.00%) | 0 ( 0.00%) | 0 ( 0.00%) |
|  | Complication associated with device | 1 ( 0.0%) | 0 ( 0.00%) | 1 ( 0.2%) | 0 ( 0.00%) | 0 ( 0.00%) |
|  | Discomfort | 1 ( 0.0%) | 0 ( 0.00%) | 1 ( 0.2%) | 0 ( 0.00%) | 0 ( 0.00%) |
|  | Drug effect incomplete | 1 ( 0.0%) | 0 ( 0.00%) | 0 ( 0.00%) | 1 ( 0.2%) | 0 ( 0.00%) |
|  | Fibrosis | 1 ( 0.0%) | 0 ( 0.00%) | 0 ( 0.00%) | 0 ( 0.00%) | 1 ( 0.5%) |
|  | Foreign body reaction | 1 ( 0.0%) | 1 ( 0.2%) | 0 ( 0.00%) | 0 ( 0.00%) | 0 ( 0.00%) |
|  | Impaired healing | 1 ( 0.0%) | 0 ( 0.00%) | 1 ( 0.2%) | 0 ( 0.00%) | 0 ( 0.00%) |
|  | Inflammation | 1 ( 0.0%) | 1 ( 0.2%) | 0 ( 0.00%) | 0 ( 0.00%) | 0 ( 0.00%) |
|  | Influenza like illness | 1 ( 0.0%) | 1 ( 0.2%) | 0 ( 0.00%) | 0 ( 0.00%) | 0 ( 0.00%) |
|  | Injection site irritation | 1 ( 0.0%) | 0 ( 0.00%) | 0 ( 0.00%) | 1 ( 0.2%) | 0 ( 0.00%) |
|  | Injection site reaction | 1 ( 0.0%) | 0 ( 0.00%) | 0 ( 0.00%) | 1 ( 0.2%) | 0 ( 0.00%) |
|  | Necrosis | 1 ( 0.0%) | 1 ( 0.2%) | 0 ( 0.00%) | 0 ( 0.00%) | 0 ( 0.00%) |
|  | No adverse event | 1 ( 0.0%) | 0 ( 0.00%) | 1 ( 0.2%) | 0 ( 0.00%) | 0 ( 0.00%) |
|  | Polyp | 1 ( 0.0%) | 0 ( 0.00%) | 1 ( 0.2%) | 0 ( 0.00%) | 0 ( 0.00%) |
|  | Pyrexia | 1 ( 0.0%) | 0 ( 0.00%) | 1 ( 0.2%) | 0 ( 0.00%) | 0 ( 0.00%) |
|  | Sensation of foreign body | 1 ( 0.0%) | 1 ( 0.2%) | 0 ( 0.00%) | 0 ( 0.00%) | 0 ( 0.00%) |
|  | Swelling | 1 ( 0.0%) | 0 ( 0.00%) | 0 ( 0.00%) | 0 ( 0.00%) | 1 ( 0.5%) |
|  | Ulcer | 1 ( 0.0%) | 0 ( 0.00%) | 1 ( 0.2%) | 0 ( 0.00%) | 0 ( 0.00%) |
|  |  |  |  |  |  |  |
| Investigations | All Patients | 181 ( 3.1%) | 73 ( 16.2%) | 27 ( 6.5%) | 79 ( 15.9%) | 13 ( 6.1%) |
|  | Intraocular pressure increased | 144 ( 2.5%) | 65 ( 14.4%) | 14 ( 3.4%) | 65 ( 13.1%) | 9 ( 4.2%) |
|  | Angiogram abnormal | 5 ( 0.1%) | 0 ( 0.00%) | 1 ( 0.2%) | 4 ( 0.8%) | 0 ( 0.00%) |
|  | Intraocular pressure decreased | 5 ( 0.1%) | 0 ( 0.00%) | 0 ( 0.00%) | 5 ( 1.0%) | 0 ( 0.00%) |
|  | Intraocular pressure test abnormal | 5 ( 0.1%) | 3 ( 0.7%) | 1 ( 0.2%) | 0 ( 0.00%) | 1 ( 0.5%) |
|  | Glycosylated haemoglobin increased | 4 ( 0.1%) | 1 ( 0.2%) | 0 ( 0.00%) | 3 ( 0.6%) | 0 ( 0.00%) |
|  | Blood pressure abnormal | 2 ( 0.0%) | 0 ( 0.00%) | 1 ( 0.2%) | 1 ( 0.2%) | 0 ( 0.00%) |
|  | Blood pressure systolic increased | 2 ( 0.0%) | 0 ( 0.00%) | 0 ( 0.00%) | 0 ( 0.00%) | 2 ( 0.9%) |
|  | Catheterisation cardiac | 2 ( 0.0%) | 1 ( 0.2%) | 1 ( 0.2%) | 0 ( 0.00%) | 0 ( 0.00%) |
|  | International normalised ratio increased | 2 ( 0.0%) | 0 ( 0.00%) | 1 ( 0.2%) | 1 ( 0.2%) | 0 ( 0.00%) |
|  | Intraocular pressure fluctuation | 2 ( 0.0%) | 1 ( 0.2%) | 1 ( 0.2%) | 0 ( 0.00%) | 0 ( 0.00%) |
|  | Angiogram retina abnormal | 1 ( 0.0%) | 0 ( 0.00%) | 0 ( 0.00%) | 1 ( 0.2%) | 0 ( 0.00%) |
|  | Biopsy liver | 1 ( 0.0%) | 0 ( 0.00%) | 1 ( 0.2%) | 0 ( 0.00%) | 0 ( 0.00%) |
|  | Blood cholesterol increased | 1 ( 0.0%) | 1 ( 0.2%) | 0 ( 0.00%) | 0 ( 0.00%) | 0 ( 0.00%) |
|  | Blood creatine phosphokinase increased | 1 ( 0.0%) | 0 ( 0.00%) | 0 ( 0.00%) | 1 ( 0.2%) | 0 ( 0.00%) |
|  | Blood creatinine increased | 1 ( 0.0%) | 1 ( 0.2%) | 0 ( 0.00%) | 0 ( 0.00%) | 0 ( 0.00%) |
|  | Blood culture positive | 1 ( 0.0%) | 0 ( 0.00%) | 1 ( 0.2%) | 0 ( 0.00%) | 0 ( 0.00%) |
|  | Blood lactic acid increased | 1 ( 0.0%) | 0 ( 0.00%) | 0 ( 0.00%) | 1 ( 0.2%) | 0 ( 0.00%) |
|  | Blood pressure increased | 1 ( 0.0%) | 0 ( 0.00%) | 1 ( 0.2%) | 0 ( 0.00%) | 0 ( 0.00%) |
|  | Blood thyroid stimulating hormone decreased | 1 ( 0.0%) | 0 ( 0.00%) | 0 ( 0.00%) | 1 ( 0.2%) | 0 ( 0.00%) |
|  | C-reactive protein increased | 1 ( 0.0%) | 0 ( 0.00%) | 0 ( 0.00%) | 1 ( 0.2%) | 0 ( 0.00%) |
|  | Cystoscopy | 1 ( 0.0%) | 1 ( 0.2%) | 0 ( 0.00%) | 0 ( 0.00%) | 0 ( 0.00%) |
|  | Full blood count increased | 1 ( 0.0%) | 0 ( 0.00%) | 1 ( 0.2%) | 0 ( 0.00%) | 0 ( 0.00%) |
|  | General physical condition abnormal | 1 ( 0.0%) | 1 ( 0.2%) | 0 ( 0.00%) | 0 ( 0.00%) | 0 ( 0.00%) |
|  | Glycosylated haemoglobin | 1 ( 0.0%) | 0 ( 0.00%) | 0 ( 0.00%) | 0 ( 0.00%) | 1 ( 0.5%) |
|  | Haemoglobin decreased | 1 ( 0.0%) | 0 ( 0.00%) | 1 ( 0.2%) | 0 ( 0.00%) | 0 ( 0.00%) |
|  | Hepatic enzyme increased | 1 ( 0.0%) | 0 ( 0.00%) | 1 ( 0.2%) | 0 ( 0.00%) | 0 ( 0.00%) |
|  | Intraocular pressure test | 1 ( 0.0%) | 0 ( 0.00%) | 1 ( 0.2%) | 0 ( 0.00%) | 0 ( 0.00%) |
|  | Norovirus test positive | 1 ( 0.0%) | 0 ( 0.00%) | 1 ( 0.2%) | 0 ( 0.00%) | 0 ( 0.00%) |
|  | Visual acuity tests abnormal | 1 ( 0.0%) | 0 ( 0.00%) | 0 ( 0.00%) | 1 ( 0.2%) | 0 ( 0.00%) |
|  | Weight decreased | 1 ( 0.0%) | 0 ( 0.00%) | 1 ( 0.2%) | 0 ( 0.00%) | 0 ( 0.00%) |
|  | White blood cell count decreased | 1 ( 0.0%) | 1 ( 0.2%) | 0 ( 0.00%) | 0 ( 0.00%) | 0 ( 0.00%) |
|  |  |  |  |  |  |  |
| Surgical and medical procedures | All Patients | 153 ( 2.6%) | 90 ( 20.0%) | 29 ( 7.0%) | 22 ( 4.4%) | 15 ( 7.0%) |
|  | Cataract operation | 75 ( 1.3%) | 57 ( 12.6%) | 5 ( 1.2%) | 13 ( 2.6%) | 0 ( 0.00%) |
|  | Drug therapy | 14 ( 0.2%) | 9 ( 2.0%) | 2 ( 0.5%) | 4 ( 0.8%) | 0 ( 0.00%) |
|  | Hospitalisation | 12 ( 0.2%) | 1 ( 0.2%) | 3 ( 0.7%) | 0 ( 0.00%) | 9 ( 4.2%) |
|  | Laser therapy | 11 ( 0.2%) | 7 ( 1.6%) | 0 ( 0.00%) | 4 ( 0.8%) | 0 ( 0.00%) |
|  | Vitrectomy | 10 ( 0.2%) | 3 ( 0.7%) | 6 ( 1.4%) | 0 ( 0.00%) | 1 ( 0.5%) |
|  | Therapy change | 5 ( 0.1%) | 5 ( 1.1%) | 0 ( 0.00%) | 0 ( 0.00%) | 0 ( 0.00%) |
|  | Hip surgery | 3 ( 0.1%) | 1 ( 0.2%) | 2 ( 0.5%) | 0 ( 0.00%) | 0 ( 0.00%) |
|  | Intraocular lens implant | 3 ( 0.1%) | 2 ( 0.4%) | 1 ( 0.2%) | 0 ( 0.00%) | 0 ( 0.00%) |
|  | Knee operation | 3 ( 0.1%) | 2 ( 0.4%) | 0 ( 0.00%) | 0 ( 0.00%) | 1 ( 0.5%) |
|  | Prophylaxis | 3 ( 0.1%) | 2 ( 0.4%) | 0 ( 0.00%) | 1 ( 0.2%) | 0 ( 0.00%) |
|  | Cardiac pacemaker insertion | 2 ( 0.0%) | 1 ( 0.2%) | 1 ( 0.2%) | 0 ( 0.00%) | 0 ( 0.00%) |
|  | Heart valve operation | 2 ( 0.0%) | 0 ( 0.00%) | 2 ( 0.5%) | 0 ( 0.00%) | 0 ( 0.00%) |
|  | Lens extraction | 2 ( 0.0%) | 1 ( 0.2%) | 1 ( 0.2%) | 0 ( 0.00%) | 0 ( 0.00%) |
|  | Toe amputation | 2 ( 0.0%) | 0 ( 0.00%) | 2 ( 0.5%) | 0 ( 0.00%) | 0 ( 0.00%) |
|  | Vascular graft | 2 ( 0.0%) | 0 ( 0.00%) | 1 ( 0.2%) | 0 ( 0.00%) | 1 ( 0.5%) |
|  | Arterial repair | 1 ( 0.0%) | 0 ( 0.00%) | 1 ( 0.2%) | 0 ( 0.00%) | 0 ( 0.00%) |
|  | Blepharoplasty | 1 ( 0.0%) | 1 ( 0.2%) | 0 ( 0.00%) | 0 ( 0.00%) | 0 ( 0.00%) |
|  | Bunion operation | 1 ( 0.0%) | 1 ( 0.2%) | 0 ( 0.00%) | 0 ( 0.00%) | 0 ( 0.00%) |
|  | Cardiac operation | 1 ( 0.0%) | 0 ( 0.00%) | 0 ( 0.00%) | 0 ( 0.00%) | 1 ( 0.5%) |
|  | Cholecystectomy | 1 ( 0.0%) | 0 ( 0.00%) | 1 ( 0.2%) | 0 ( 0.00%) | 0 ( 0.00%) |
|  | Ciliary body operation | 1 ( 0.0%) | 0 ( 0.00%) | 1 ( 0.2%) | 0 ( 0.00%) | 0 ( 0.00%) |
|  | Coronary artery bypass | 1 ( 0.0%) | 1 ( 0.2%) | 0 ( 0.00%) | 0 ( 0.00%) | 0 ( 0.00%) |
|  | Dental implantation | 1 ( 0.0%) | 1 ( 0.2%) | 0 ( 0.00%) | 0 ( 0.00%) | 0 ( 0.00%) |
|  | Elective surgery | 1 ( 0.0%) | 1 ( 0.2%) | 0 ( 0.00%) | 0 ( 0.00%) | 0 ( 0.00%) |
|  | Eye operation | 1 ( 0.0%) | 0 ( 0.00%) | 1 ( 0.2%) | 0 ( 0.00%) | 0 ( 0.00%) |
|  | Hernia repair | 1 ( 0.0%) | 0 ( 0.00%) | 1 ( 0.2%) | 0 ( 0.00%) | 0 ( 0.00%) |
|  | Hysterectomy | 1 ( 0.0%) | 0 ( 0.00%) | 1 ( 0.2%) | 0 ( 0.00%) | 0 ( 0.00%) |
|  | Iridotomy | 1 ( 0.0%) | 0 ( 0.00%) | 0 ( 0.00%) | 1 ( 0.2%) | 0 ( 0.00%) |
|  | Knee arthroplasty | 1 ( 0.0%) | 0 ( 0.00%) | 1 ( 0.2%) | 0 ( 0.00%) | 0 ( 0.00%) |
|  | Ophthalmologic treatment | 1 ( 0.0%) | 0 ( 0.00%) | 1 ( 0.2%) | 0 ( 0.00%) | 0 ( 0.00%) |
|  | Oxygen supplementation | 1 ( 0.0%) | 0 ( 0.00%) | 0 ( 0.00%) | 0 ( 0.00%) | 1 ( 0.5%) |
|  | Radiotherapy | 1 ( 0.0%) | 0 ( 0.00%) | 1 ( 0.2%) | 0 ( 0.00%) | 0 ( 0.00%) |
|  | Retinal operation | 1 ( 0.0%) | 1 ( 0.2%) | 0 ( 0.00%) | 0 ( 0.00%) | 0 ( 0.00%) |
|  | Shoulder operation | 1 ( 0.0%) | 0 ( 0.00%) | 0 ( 0.00%) | 0 ( 0.00%) | 1 ( 0.5%) |
|  | Spinal cord operation | 1 ( 0.0%) | 0 ( 0.00%) | 1 ( 0.2%) | 0 ( 0.00%) | 0 ( 0.00%) |
|  | Spinal operation | 1 ( 0.0%) | 0 ( 0.00%) | 1 ( 0.2%) | 0 ( 0.00%) | 0 ( 0.00%) |
|  | Suture fixation of intraocular lens | 1 ( 0.0%) | 0 ( 0.00%) | 1 ( 0.2%) | 0 ( 0.00%) | 0 ( 0.00%) |
|  | Suture removal | 1 ( 0.0%) | 1 ( 0.2%) | 0 ( 0.00%) | 0 ( 0.00%) | 0 ( 0.00%) |
|  | Tendon operation | 1 ( 0.0%) | 1 ( 0.2%) | 0 ( 0.00%) | 0 ( 0.00%) | 0 ( 0.00%) |
|  | Therapeutic procedure | 1 ( 0.0%) | 0 ( 0.00%) | 1 ( 0.2%) | 0 ( 0.00%) | 0 ( 0.00%) |
|  | Tooth extraction | 1 ( 0.0%) | 1 ( 0.2%) | 0 ( 0.00%) | 0 ( 0.00%) | 0 ( 0.00%) |
|  |  |  |  |  |  |  |
| Infections and infestations | All Patients | 130 ( 2.2%) | 68 ( 15.1%) | 46 ( 11.0%) | 12 ( 2.4%) | 9 ( 4.2%) |
|  | Conjunctivitis | 29 ( 0.5%) | 25 ( 5.5%) | 0 ( 0.00%) | 4 ( 0.8%) | 0 ( 0.00%) |
|  | Pneumonia | 27 ( 0.5%) | 2 ( 0.4%) | 22 ( 5.3%) | 1 ( 0.2%) | 2 ( 0.9%) |
|  | Nasopharyngitis | 20 ( 0.3%) | 17 ( 3.8%) | 0 ( 0.00%) | 3 ( 0.6%) | 0 ( 0.00%) |
|  | Influenza | 9 ( 0.2%) | 7 ( 1.6%) | 1 ( 0.2%) | 1 ( 0.2%) | 0 ( 0.00%) |
|  | Endophthalmitis | 7 ( 0.1%) | 0 ( 0.00%) | 1 ( 0.2%) | 0 ( 0.00%) | 6 ( 2.8%) |
|  | Hordeolum | 6 ( 0.1%) | 5 ( 1.1%) | 1 ( 0.2%) | 0 ( 0.00%) | 0 ( 0.00%) |
|  | Sepsis | 5 ( 0.1%) | 0 ( 0.00%) | 5 ( 1.2%) | 0 ( 0.00%) | 0 ( 0.00%) |
|  | Ophthalmic herpes simplex | 4 ( 0.1%) | 1 ( 0.2%) | 1 ( 0.2%) | 1 ( 0.2%) | 1 ( 0.5%) |
|  | Respiratory tract infection | 4 ( 0.1%) | 1 ( 0.2%) | 3 ( 0.7%) | 0 ( 0.00%) | 0 ( 0.00%) |
|  | Bronchitis | 3 ( 0.1%) | 3 ( 0.7%) | 0 ( 0.00%) | 0 ( 0.00%) | 0 ( 0.00%) |
|  | Gastroenteritis | 3 ( 0.1%) | 3 ( 0.7%) | 0 ( 0.00%) | 0 ( 0.00%) | 0 ( 0.00%) |
|  | Herpes zoster | 3 ( 0.1%) | 3 ( 0.7%) | 0 ( 0.00%) | 0 ( 0.00%) | 0 ( 0.00%) |
|  | Infection | 3 ( 0.1%) | 2 ( 0.4%) | 1 ( 0.2%) | 0 ( 0.00%) | 0 ( 0.00%) |
|  | Febrile infection | 2 ( 0.0%) | 0 ( 0.00%) | 2 ( 0.5%) | 0 ( 0.00%) | 0 ( 0.00%) |
|  | Pyelitis | 2 ( 0.0%) | 0 ( 0.00%) | 2 ( 0.5%) | 0 ( 0.00%) | 0 ( 0.00%) |
|  | Abscess | 1 ( 0.0%) | 0 ( 0.00%) | 0 ( 0.00%) | 1 ( 0.2%) | 0 ( 0.00%) |
|  | Bacterial infection | 1 ( 0.0%) | 0 ( 0.00%) | 1 ( 0.2%) | 0 ( 0.00%) | 0 ( 0.00%) |
|  | Cellulitis | 1 ( 0.0%) | 0 ( 0.00%) | 1 ( 0.2%) | 0 ( 0.00%) | 0 ( 0.00%) |
|  | Conjunctivitis bacterial | 1 ( 0.0%) | 1 ( 0.2%) | 0 ( 0.00%) | 0 ( 0.00%) | 0 ( 0.00%) |
|  | Cystitis | 1 ( 0.0%) | 1 ( 0.2%) | 0 ( 0.00%) | 0 ( 0.00%) | 0 ( 0.00%) |
|  | Diabetic foot infection | 1 ( 0.0%) | 0 ( 0.00%) | 1 ( 0.2%) | 0 ( 0.00%) | 0 ( 0.00%) |
|  | Ear infection | 1 ( 0.0%) | 1 ( 0.2%) | 0 ( 0.00%) | 0 ( 0.00%) | 0 ( 0.00%) |
|  | Endocarditis | 1 ( 0.0%) | 0 ( 0.00%) | 0 ( 0.00%) | 0 ( 0.00%) | 1 ( 0.5%) |
|  | Epididymitis | 1 ( 0.0%) | 1 ( 0.2%) | 0 ( 0.00%) | 0 ( 0.00%) | 0 ( 0.00%) |
|  | Erysipelas | 1 ( 0.0%) | 0 ( 0.00%) | 1 ( 0.2%) | 0 ( 0.00%) | 0 ( 0.00%) |
|  | Gastrointestinal fungal infection | 1 ( 0.0%) | 1 ( 0.2%) | 0 ( 0.00%) | 0 ( 0.00%) | 0 ( 0.00%) |
|  | Gastrointestinal infection | 1 ( 0.0%) | 1 ( 0.2%) | 0 ( 0.00%) | 0 ( 0.00%) | 0 ( 0.00%) |
|  | Gingivitis | 1 ( 0.0%) | 1 ( 0.2%) | 0 ( 0.00%) | 0 ( 0.00%) | 0 ( 0.00%) |
|  | Helicobacter infection | 1 ( 0.0%) | 1 ( 0.2%) | 0 ( 0.00%) | 0 ( 0.00%) | 0 ( 0.00%) |
|  | Hypopyon | 1 ( 0.0%) | 0 ( 0.00%) | 1 ( 0.2%) | 0 ( 0.00%) | 0 ( 0.00%) |
|  | Lyme disease | 1 ( 0.0%) | 0 ( 0.00%) | 1 ( 0.2%) | 0 ( 0.00%) | 0 ( 0.00%) |
|  | Nipple infection | 1 ( 0.0%) | 1 ( 0.2%) | 0 ( 0.00%) | 0 ( 0.00%) | 0 ( 0.00%) |
|  | Oesophageal candidiasis | 1 ( 0.0%) | 0 ( 0.00%) | 0 ( 0.00%) | 1 ( 0.2%) | 0 ( 0.00%) |
|  | Oral herpes | 1 ( 0.0%) | 0 ( 0.00%) | 0 ( 0.00%) | 1 ( 0.2%) | 0 ( 0.00%) |
|  | Perirectal abscess | 1 ( 0.0%) | 0 ( 0.00%) | 1 ( 0.2%) | 0 ( 0.00%) | 0 ( 0.00%) |
|  | Pulpitis dental | 1 ( 0.0%) | 1 ( 0.2%) | 0 ( 0.00%) | 0 ( 0.00%) | 0 ( 0.00%) |
|  | Purulence | 1 ( 0.0%) | 1 ( 0.2%) | 0 ( 0.00%) | 0 ( 0.00%) | 0 ( 0.00%) |
|  | Rotavirus infection | 1 ( 0.0%) | 0 ( 0.00%) | 1 ( 0.2%) | 0 ( 0.00%) | 0 ( 0.00%) |
|  | Septic shock | 1 ( 0.0%) | 0 ( 0.00%) | 0 ( 0.00%) | 0 ( 0.00%) | 1 ( 0.5%) |
|  | Sinusitis | 1 ( 0.0%) | 1 ( 0.2%) | 0 ( 0.00%) | 0 ( 0.00%) | 0 ( 0.00%) |
|  | Tonsillitis | 1 ( 0.0%) | 1 ( 0.2%) | 0 ( 0.00%) | 0 ( 0.00%) | 0 ( 0.00%) |
|  | Urosepsis | 1 ( 0.0%) | 0 ( 0.00%) | 1 ( 0.2%) | 0 ( 0.00%) | 0 ( 0.00%) |
|  | Wound infection | 1 ( 0.0%) | 0 ( 0.00%) | 0 ( 0.00%) | 1 ( 0.2%) | 0 ( 0.00%) |
|  | Wound infection bacterial | 1 ( 0.0%) | 0 ( 0.00%) | 1 ( 0.2%) | 0 ( 0.00%) | 0 ( 0.00%) |
|  | Wound sepsis | 1 ( 0.0%) | 0 ( 0.00%) | 1 ( 0.2%) | 0 ( 0.00%) | 0 ( 0.00%) |
|  |  |  |  |  |  |  |
| Injury, poisoning and procedural complications | All Patients | 106 ( 1.8%) | 23 ( 5.1%) | 50 ( 12.0%) | 29 ( 5.8%) | 12 ( 5.6%) |
|  | Fall | 43 ( 0.7%) | 11 ( 2.4%) | 24 ( 5.8%) | 2 ( 0.4%) | 6 ( 2.8%) |
|  | Inappropriate schedule of drug administration | 18 ( 0.3%) | 2 ( 0.4%) | 0 ( 0.00%) | 16 ( 3.2%) | 0 ( 0.00%) |
|  | Femoral neck fracture | 14 ( 0.2%) | 0 ( 0.00%) | 13 ( 3.1%) | 0 ( 0.00%) | 1 ( 0.5%) |
|  | Spinal fracture | 4 ( 0.1%) | 0 ( 0.00%) | 4 ( 1.0%) | 0 ( 0.00%) | 0 ( 0.00%) |
|  | Upper limb fracture | 4 ( 0.1%) | 3 ( 0.7%) | 1 ( 0.2%) | 0 ( 0.00%) | 1 ( 0.5%) |
|  | Drug administered at inappropriate site | 3 ( 0.1%) | 0 ( 0.00%) | 0 ( 0.00%) | 3 ( 0.6%) | 0 ( 0.00%) |
|  | Ligament sprain | 3 ( 0.1%) | 3 ( 0.7%) | 0 ( 0.00%) | 0 ( 0.00%) | 0 ( 0.00%) |
|  | Multiple use of single-use product | 3 ( 0.1%) | 0 ( 0.00%) | 0 ( 0.00%) | 3 ( 0.6%) | 0 ( 0.00%) |
|  | Accident | 2 ( 0.0%) | 0 ( 0.00%) | 2 ( 0.5%) | 0 ( 0.00%) | 0 ( 0.00%) |
|  | Craniocerebral injury | 2 ( 0.0%) | 0 ( 0.00%) | 2 ( 0.5%) | 0 ( 0.00%) | 0 ( 0.00%) |
|  | Eyelid injury | 2 ( 0.0%) | 2 ( 0.4%) | 0 ( 0.00%) | 0 ( 0.00%) | 0 ( 0.00%) |
|  | Fracture | 2 ( 0.0%) | 0 ( 0.00%) | 1 ( 0.2%) | 0 ( 0.00%) | 1 ( 0.5%) |
|  | Lower limb fracture | 2 ( 0.0%) | 0 ( 0.00%) | 2 ( 0.5%) | 0 ( 0.00%) | 0 ( 0.00%) |
|  | Lumbar vertebral fracture | 2 ( 0.0%) | 0 ( 0.00%) | 2 ( 0.5%) | 0 ( 0.00%) | 0 ( 0.00%) |
|  | Overdose | 2 ( 0.0%) | 0 ( 0.00%) | 0 ( 0.00%) | 2 ( 0.4%) | 0 ( 0.00%) |
|  | Rib fracture | 2 ( 0.0%) | 0 ( 0.00%) | 2 ( 0.5%) | 0 ( 0.00%) | 0 ( 0.00%) |
|  | Wound | 2 ( 0.0%) | 0 ( 0.00%) | 1 ( 0.2%) | 1 ( 0.2%) | 0 ( 0.00%) |
|  | Arthropod bite | 1 ( 0.0%) | 1 ( 0.2%) | 0 ( 0.00%) | 0 ( 0.00%) | 0 ( 0.00%) |
|  | Cardiac valve rupture | 1 ( 0.0%) | 0 ( 0.00%) | 0 ( 0.00%) | 0 ( 0.00%) | 1 ( 0.5%) |
|  | Contusion | 1 ( 0.0%) | 1 ( 0.2%) | 0 ( 0.00%) | 0 ( 0.00%) | 0 ( 0.00%) |
|  | Drug administration error | 1 ( 0.0%) | 0 ( 0.00%) | 0 ( 0.00%) | 1 ( 0.2%) | 0 ( 0.00%) |
|  | Eye burns | 1 ( 0.0%) | 1 ( 0.2%) | 0 ( 0.00%) | 0 ( 0.00%) | 0 ( 0.00%) |
|  | Eye contusion | 1 ( 0.0%) | 0 ( 0.00%) | 0 ( 0.00%) | 0 ( 0.00%) | 1 ( 0.5%) |
|  | Eye injury | 1 ( 0.0%) | 1 ( 0.2%) | 0 ( 0.00%) | 0 ( 0.00%) | 0 ( 0.00%) |
|  | Eye laser scar | 1 ( 0.0%) | 1 ( 0.2%) | 0 ( 0.00%) | 0 ( 0.00%) | 0 ( 0.00%) |
|  | Facial bones fracture | 1 ( 0.0%) | 0 ( 0.00%) | 1 ( 0.2%) | 0 ( 0.00%) | 0 ( 0.00%) |
|  | Femur fracture | 1 ( 0.0%) | 0 ( 0.00%) | 1 ( 0.2%) | 0 ( 0.00%) | 0 ( 0.00%) |
|  | Foot fracture | 1 ( 0.0%) | 0 ( 0.00%) | 1 ( 0.2%) | 0 ( 0.00%) | 0 ( 0.00%) |
|  | Injury corneal | 1 ( 0.0%) | 1 ( 0.2%) | 0 ( 0.00%) | 0 ( 0.00%) | 0 ( 0.00%) |
|  | Open globe injury | 1 ( 0.0%) | 0 ( 0.00%) | 1 ( 0.2%) | 0 ( 0.00%) | 0 ( 0.00%) |
|  | Pelvic fracture | 1 ( 0.0%) | 0 ( 0.00%) | 1 ( 0.2%) | 0 ( 0.00%) | 0 ( 0.00%) |
|  | Periorbital haematoma | 1 ( 0.0%) | 0 ( 0.00%) | 1 ( 0.2%) | 0 ( 0.00%) | 0 ( 0.00%) |
|  | Post procedural haematoma | 1 ( 0.0%) | 0 ( 0.00%) | 1 ( 0.2%) | 0 ( 0.00%) | 0 ( 0.00%) |
|  | Prescribed overdose | 1 ( 0.0%) | 0 ( 0.00%) | 0 ( 0.00%) | 1 ( 0.2%) | 0 ( 0.00%) |
|  | Procedural pain | 1 ( 0.0%) | 1 ( 0.2%) | 0 ( 0.00%) | 0 ( 0.00%) | 0 ( 0.00%) |
|  | Retinal injury | 1 ( 0.0%) | 0 ( 0.00%) | 0 ( 0.00%) | 0 ( 0.00%) | 1 ( 0.5%) |
|  | Scar | 1 ( 0.0%) | 0 ( 0.00%) | 1 ( 0.2%) | 0 ( 0.00%) | 0 ( 0.00%) |
|  | Skin injury | 1 ( 0.0%) | 0 ( 0.00%) | 1 ( 0.2%) | 0 ( 0.00%) | 0 ( 0.00%) |
|  | Spinal column injury | 1 ( 0.0%) | 0 ( 0.00%) | 1 ( 0.2%) | 0 ( 0.00%) | 0 ( 0.00%) |
|  | Spinal cord injury cervical | 1 ( 0.0%) | 0 ( 0.00%) | 1 ( 0.2%) | 0 ( 0.00%) | 0 ( 0.00%) |
|  | Subarachnoid haemorrhage | 1 ( 0.0%) | 0 ( 0.00%) | 1 ( 0.2%) | 0 ( 0.00%) | 0 ( 0.00%) |
|  | Subdural haematoma | 1 ( 0.0%) | 0 ( 0.00%) | 1 ( 0.2%) | 0 ( 0.00%) | 0 ( 0.00%) |
|  | Thoracic vertebral fracture | 1 ( 0.0%) | 0 ( 0.00%) | 1 ( 0.2%) | 0 ( 0.00%) | 0 ( 0.00%) |
|  | Toxic anterior segment syndrome | 1 ( 0.0%) | 0 ( 0.00%) | 0 ( 0.00%) | 0 ( 0.00%) | 1 ( 0.5%) |
|  | Traumatic haematoma | 1 ( 0.0%) | 1 ( 0.2%) | 0 ( 0.00%) | 0 ( 0.00%) | 0 ( 0.00%) |
|  |  |  |  |  |  |  |
| Nervous system disorders | All Patients | 85 ( 1.5%) | 12 ( 2.7%) | 38 ( 9.1%) | 7 ( 1.4%) | 30 ( 14.1%) |
|  | Cerebrovascular accident | 27 ( 0.5%) | 0 ( 0.00%) | 11 ( 2.6%) | 0 ( 0.00%) | 16 ( 7.5%) |
|  | Dizziness | 10 ( 0.2%) | 2 ( 0.4%) | 4 ( 1.0%) | 1 ( 0.2%) | 3 ( 1.4%) |
|  | Motor dysfunction | 8 ( 0.1%) | 0 ( 0.00%) | 3 ( 0.7%) | 0 ( 0.00%) | 5 ( 2.3%) |
|  | Aphasia | 5 ( 0.1%) | 0 ( 0.00%) | 2 ( 0.5%) | 0 ( 0.00%) | 3 ( 1.4%) |
|  | Coordination abnormal | 5 ( 0.1%) | 0 ( 0.00%) | 4 ( 1.0%) | 0 ( 0.00%) | 1 ( 0.5%) |
|  | Dementia | 4 ( 0.1%) | 0 ( 0.00%) | 3 ( 0.7%) | 0 ( 0.00%) | 1 ( 0.5%) |
|  | Headache | 4 ( 0.1%) | 2 ( 0.4%) | 0 ( 0.00%) | 0 ( 0.00%) | 2 ( 0.9%) |
|  | Syncope | 4 ( 0.1%) | 1 ( 0.2%) | 3 ( 0.7%) | 0 ( 0.00%) | 0 ( 0.00%) |
|  | Visual field defect | 4 ( 0.1%) | 0 ( 0.00%) | 1 ( 0.2%) | 2 ( 0.4%) | 1 ( 0.5%) |
|  | Loss of consciousness | 3 ( 0.1%) | 0 ( 0.00%) | 1 ( 0.2%) | 0 ( 0.00%) | 2 ( 0.9%) |
|  | Paralysis | 3 ( 0.1%) | 0 ( 0.00%) | 3 ( 0.7%) | 0 ( 0.00%) | 0 ( 0.00%) |
|  | Balance disorder | 2 ( 0.0%) | 0 ( 0.00%) | 1 ( 0.2%) | 0 ( 0.00%) | 1 ( 0.5%) |
|  | Carotid artery stenosis | 2 ( 0.0%) | 0 ( 0.00%) | 2 ( 0.5%) | 0 ( 0.00%) | 0 ( 0.00%) |
|  | Cerebral infarction | 2 ( 0.0%) | 0 ( 0.00%) | 0 ( 0.00%) | 0 ( 0.00%) | 2 ( 0.9%) |
|  | Diabetic neuropathy | 2 ( 0.0%) | 0 ( 0.00%) | 2 ( 0.5%) | 0 ( 0.00%) | 0 ( 0.00%) |
|  | Hypoaesthesia | 2 ( 0.0%) | 0 ( 0.00%) | 1 ( 0.2%) | 1 ( 0.2%) | 0 ( 0.00%) |
|  | Ischaemic stroke | 2 ( 0.0%) | 0 ( 0.00%) | 0 ( 0.00%) | 0 ( 0.00%) | 2 ( 0.9%) |
|  | Parkinson's disease | 2 ( 0.0%) | 0 ( 0.00%) | 2 ( 0.5%) | 0 ( 0.00%) | 0 ( 0.00%) |
|  | Senile dementia | 2 ( 0.0%) | 0 ( 0.00%) | 1 ( 0.2%) | 1 ( 0.2%) | 0 ( 0.00%) |
|  | Spinal cord haemorrhage | 2 ( 0.0%) | 0 ( 0.00%) | 1 ( 0.2%) | 0 ( 0.00%) | 1 ( 0.5%) |
|  | Transient ischaemic attack | 2 ( 0.0%) | 0 ( 0.00%) | 0 ( 0.00%) | 0 ( 0.00%) | 2 ( 0.9%) |
|  | Tremor | 2 ( 0.0%) | 1 ( 0.2%) | 0 ( 0.00%) | 1 ( 0.2%) | 0 ( 0.00%) |
|  | Carotid artery thrombosis | 1 ( 0.0%) | 0 ( 0.00%) | 1 ( 0.2%) | 0 ( 0.00%) | 0 ( 0.00%) |
|  | Cerebral haemorrhage | 1 ( 0.0%) | 0 ( 0.00%) | 1 ( 0.2%) | 0 ( 0.00%) | 0 ( 0.00%) |
|  | Cerebral hypoperfusion | 1 ( 0.0%) | 0 ( 0.00%) | 1 ( 0.2%) | 0 ( 0.00%) | 0 ( 0.00%) |
|  | Cerebral ischaemia | 1 ( 0.0%) | 0 ( 0.00%) | 0 ( 0.00%) | 0 ( 0.00%) | 1 ( 0.5%) |
|  | Dementia Alzheimer's type | 1 ( 0.0%) | 0 ( 0.00%) | 1 ( 0.2%) | 0 ( 0.00%) | 0 ( 0.00%) |
|  | Embolic stroke | 1 ( 0.0%) | 0 ( 0.00%) | 1 ( 0.2%) | 0 ( 0.00%) | 0 ( 0.00%) |
|  | Epilepsy | 1 ( 0.0%) | 0 ( 0.00%) | 1 ( 0.2%) | 0 ( 0.00%) | 0 ( 0.00%) |
|  | Facial paralysis | 1 ( 0.0%) | 1 ( 0.2%) | 0 ( 0.00%) | 0 ( 0.00%) | 0 ( 0.00%) |
|  | Gliosis | 1 ( 0.0%) | 0 ( 0.00%) | 0 ( 0.00%) | 1 ( 0.2%) | 0 ( 0.00%) |
|  | Hemiparesis | 1 ( 0.0%) | 0 ( 0.00%) | 0 ( 0.00%) | 0 ( 0.00%) | 1 ( 0.5%) |
|  | Hemiplegia | 1 ( 0.0%) | 0 ( 0.00%) | 1 ( 0.2%) | 0 ( 0.00%) | 0 ( 0.00%) |
|  | Hydrocephalus | 1 ( 0.0%) | 0 ( 0.00%) | 1 ( 0.2%) | 0 ( 0.00%) | 0 ( 0.00%) |
|  | IIIrd nerve paralysis | 1 ( 0.0%) | 0 ( 0.00%) | 1 ( 0.2%) | 0 ( 0.00%) | 0 ( 0.00%) |
|  | IIIrd nerve paresis | 1 ( 0.0%) | 0 ( 0.00%) | 0 ( 0.00%) | 0 ( 0.00%) | 1 ( 0.5%) |
|  | Intraventricular haemorrhage | 1 ( 0.0%) | 0 ( 0.00%) | 1 ( 0.2%) | 0 ( 0.00%) | 0 ( 0.00%) |
|  | Migraine | 1 ( 0.0%) | 1 ( 0.2%) | 0 ( 0.00%) | 0 ( 0.00%) | 0 ( 0.00%) |
|  | Mononeuritis | 1 ( 0.0%) | 0 ( 0.00%) | 0 ( 0.00%) | 0 ( 0.00%) | 1 ( 0.5%) |
|  | Myasthenia gravis | 1 ( 0.0%) | 0 ( 0.00%) | 1 ( 0.2%) | 0 ( 0.00%) | 0 ( 0.00%) |
|  | Neuralgia | 1 ( 0.0%) | 1 ( 0.2%) | 0 ( 0.00%) | 0 ( 0.00%) | 0 ( 0.00%) |
|  | Normal pressure hydrocephalus | 1 ( 0.0%) | 0 ( 0.00%) | 1 ( 0.2%) | 0 ( 0.00%) | 0 ( 0.00%) |
|  | Paraesthesia | 1 ( 0.0%) | 1 ( 0.2%) | 0 ( 0.00%) | 0 ( 0.00%) | 0 ( 0.00%) |
|  | Partial seizures | 1 ( 0.0%) | 0 ( 0.00%) | 1 ( 0.2%) | 0 ( 0.00%) | 0 ( 0.00%) |
|  | Phrenic nerve paralysis | 1 ( 0.0%) | 0 ( 0.00%) | 0 ( 0.00%) | 0 ( 0.00%) | 1 ( 0.5%) |
|  | Sciatica | 1 ( 0.0%) | 1 ( 0.2%) | 0 ( 0.00%) | 0 ( 0.00%) | 0 ( 0.00%) |
|  | Sensorimotor disorder | 1 ( 0.0%) | 0 ( 0.00%) | 1 ( 0.2%) | 0 ( 0.00%) | 0 ( 0.00%) |
|  | Sensory loss | 1 ( 0.0%) | 0 ( 0.00%) | 0 ( 0.00%) | 0 ( 0.00%) | 1 ( 0.5%) |
|  | VIth nerve paralysis | 1 ( 0.0%) | 1 ( 0.2%) | 0 ( 0.00%) | 0 ( 0.00%) | 0 ( 0.00%) |
|  |  |  |  |  |  |  |
| Cardiac disorders | All Patients | 80 ( 1.4%) | 4 ( 0.9%) | 56 ( 13.4%) | 4 ( 0.8%) | 22 ( 10.3%) |
|  | Cardiac failure | 23 ( 0.4%) | 0 ( 0.00%) | 21 ( 5.0%) | 0 ( 0.00%) | 2 ( 0.9%) |
|  | Myocardial infarction | 16 ( 0.3%) | 0 ( 0.00%) | 11 ( 2.6%) | 0 ( 0.00%) | 5 ( 2.3%) |
|  | Angina pectoris | 7 ( 0.1%) | 0 ( 0.00%) | 1 ( 0.2%) | 0 ( 0.00%) | 6 ( 2.8%) |
|  | Atrial fibrillation | 7 ( 0.1%) | 0 ( 0.00%) | 4 ( 1.0%) | 0 ( 0.00%) | 3 ( 1.4%) |
|  | Cardiac arrest | 6 ( 0.1%) | 0 ( 0.00%) | 6 ( 1.4%) | 0 ( 0.00%) | 0 ( 0.00%) |
|  | Cardiac disorder | 6 ( 0.1%) | 0 ( 0.00%) | 6 ( 1.4%) | 0 ( 0.00%) | 0 ( 0.00%) |
|  | Coronary artery disease | 6 ( 0.1%) | 0 ( 0.00%) | 3 ( 0.7%) | 0 ( 0.00%) | 3 ( 1.4%) |
|  | Arrhythmia | 5 ( 0.1%) | 0 ( 0.00%) | 2 ( 0.5%) | 0 ( 0.00%) | 3 ( 1.4%) |
|  | Cardiovascular disorder | 5 ( 0.1%) | 3 ( 0.7%) | 2 ( 0.5%) | 0 ( 0.00%) | 0 ( 0.00%) |
|  | Acute myocardial infarction | 2 ( 0.0%) | 0 ( 0.00%) | 1 ( 0.2%) | 0 ( 0.00%) | 1 ( 0.5%) |
|  | Aortic valve stenosis | 2 ( 0.0%) | 0 ( 0.00%) | 1 ( 0.2%) | 0 ( 0.00%) | 1 ( 0.5%) |
|  | Supraventricular extrasystoles | 2 ( 0.0%) | 0 ( 0.00%) | 1 ( 0.2%) | 1 ( 0.2%) | 0 ( 0.00%) |
|  | Angina unstable | 1 ( 0.0%) | 0 ( 0.00%) | 1 ( 0.2%) | 0 ( 0.00%) | 0 ( 0.00%) |
|  | Atrial flutter | 1 ( 0.0%) | 0 ( 0.00%) | 1 ( 0.2%) | 0 ( 0.00%) | 0 ( 0.00%) |
|  | Atrial tachycardia | 1 ( 0.0%) | 0 ( 0.00%) | 1 ( 0.2%) | 0 ( 0.00%) | 0 ( 0.00%) |
|  | Atrioventricular block complete | 1 ( 0.0%) | 0 ( 0.00%) | 1 ( 0.2%) | 0 ( 0.00%) | 0 ( 0.00%) |
|  | Bradyarrhythmia | 1 ( 0.0%) | 0 ( 0.00%) | 1 ( 0.2%) | 0 ( 0.00%) | 0 ( 0.00%) |
|  | Cardiac failure acute | 1 ( 0.0%) | 0 ( 0.00%) | 1 ( 0.2%) | 0 ( 0.00%) | 0 ( 0.00%) |
|  | Cardiogenic shock | 1 ( 0.0%) | 0 ( 0.00%) | 1 ( 0.2%) | 0 ( 0.00%) | 0 ( 0.00%) |
|  | Cardiopulmonary failure | 1 ( 0.0%) | 0 ( 0.00%) | 1 ( 0.2%) | 0 ( 0.00%) | 0 ( 0.00%) |
|  | Cardiorenal syndrome | 1 ( 0.0%) | 0 ( 0.00%) | 1 ( 0.2%) | 0 ( 0.00%) | 0 ( 0.00%) |
|  | Cardiovascular insufficiency | 1 ( 0.0%) | 1 ( 0.2%) | 0 ( 0.00%) | 0 ( 0.00%) | 0 ( 0.00%) |
|  | Coronary artery insufficiency | 1 ( 0.0%) | 0 ( 0.00%) | 1 ( 0.2%) | 0 ( 0.00%) | 0 ( 0.00%) |
|  | Heart valve stenosis | 1 ( 0.0%) | 0 ( 0.00%) | 0 ( 0.00%) | 0 ( 0.00%) | 1 ( 0.5%) |
|  | Left ventricular hypertrophy | 1 ( 0.0%) | 0 ( 0.00%) | 0 ( 0.00%) | 1 ( 0.2%) | 0 ( 0.00%) |
|  | Mitral valve incompetence | 1 ( 0.0%) | 0 ( 0.00%) | 0 ( 0.00%) | 1 ( 0.2%) | 0 ( 0.00%) |
|  | Myocardial ischaemia | 1 ( 0.0%) | 0 ( 0.00%) | 1 ( 0.2%) | 0 ( 0.00%) | 0 ( 0.00%) |
|  | Myocarditis | 1 ( 0.0%) | 0 ( 0.00%) | 0 ( 0.00%) | 0 ( 0.00%) | 1 ( 0.5%) |
|  | Palpitations | 1 ( 0.0%) | 0 ( 0.00%) | 0 ( 0.00%) | 1 ( 0.2%) | 0 ( 0.00%) |
|  | Sinoatrial block | 1 ( 0.0%) | 0 ( 0.00%) | 1 ( 0.2%) | 0 ( 0.00%) | 0 ( 0.00%) |
|  | Tachycardia | 1 ( 0.0%) | 0 ( 0.00%) | 0 ( 0.00%) | 1 ( 0.2%) | 0 ( 0.00%) |
|  | Tricuspid valve disease | 1 ( 0.0%) | 0 ( 0.00%) | 0 ( 0.00%) | 1 ( 0.2%) | 0 ( 0.00%) |
|  |  |  |  |  |  |  |
| Neoplasms benign, malignant and unspecified (incl cysts and polyps) | All Patients | 51 ( 0.9%) | 0 ( 0.00%) | 44 ( 10.6%) | 1 ( 0.2%) | 7 ( 3.3%) |
|  | Gastrointestinal carcinoma | 8 ( 0.1%) | 0 ( 0.00%) | 6 ( 1.4%) | 0 ( 0.00%) | 2 ( 0.9%) |
|  | Bronchial carcinoma | 3 ( 0.1%) | 0 ( 0.00%) | 2 ( 0.5%) | 0 ( 0.00%) | 1 ( 0.5%) |
|  | Lung neoplasm malignant | 3 ( 0.1%) | 0 ( 0.00%) | 2 ( 0.5%) | 0 ( 0.00%) | 1 ( 0.5%) |
|  | Metastases to liver | 3 ( 0.1%) | 0 ( 0.00%) | 3 ( 0.7%) | 0 ( 0.00%) | 0 ( 0.00%) |
|  | Neoplasm malignant | 3 ( 0.1%) | 0 ( 0.00%) | 2 ( 0.5%) | 0 ( 0.00%) | 1 ( 0.5%) |
|  | Basal cell carcinoma | 2 ( 0.0%) | 0 ( 0.00%) | 1 ( 0.2%) | 1 ( 0.2%) | 0 ( 0.00%) |
|  | Bladder cancer | 2 ( 0.0%) | 0 ( 0.00%) | 1 ( 0.2%) | 0 ( 0.00%) | 1 ( 0.5%) |
|  | Breast cancer | 2 ( 0.0%) | 0 ( 0.00%) | 2 ( 0.5%) | 0 ( 0.00%) | 0 ( 0.00%) |
|  | Colon cancer | 2 ( 0.0%) | 0 ( 0.00%) | 2 ( 0.5%) | 0 ( 0.00%) | 0 ( 0.00%) |
|  | Eyelid tumour | 2 ( 0.0%) | 0 ( 0.00%) | 2 ( 0.5%) | 0 ( 0.00%) | 0 ( 0.00%) |
|  | Hepatocellular carcinoma | 2 ( 0.0%) | 0 ( 0.00%) | 2 ( 0.5%) | 0 ( 0.00%) | 0 ( 0.00%) |
|  | Prostate cancer | 2 ( 0.0%) | 0 ( 0.00%) | 1 ( 0.2%) | 0 ( 0.00%) | 1 ( 0.5%) |
|  | Adenocarcinoma | 1 ( 0.0%) | 0 ( 0.00%) | 1 ( 0.2%) | 0 ( 0.00%) | 0 ( 0.00%) |
|  | Bile duct cancer | 1 ( 0.0%) | 0 ( 0.00%) | 1 ( 0.2%) | 0 ( 0.00%) | 0 ( 0.00%) |
|  | Brain neoplasm | 1 ( 0.0%) | 0 ( 0.00%) | 1 ( 0.2%) | 0 ( 0.00%) | 0 ( 0.00%) |
|  | Breast cancer metastatic | 1 ( 0.0%) | 0 ( 0.00%) | 1 ( 0.2%) | 0 ( 0.00%) | 0 ( 0.00%) |
|  | Cervix carcinoma | 1 ( 0.0%) | 0 ( 0.00%) | 1 ( 0.2%) | 0 ( 0.00%) | 0 ( 0.00%) |
|  | Cholangiocarcinoma | 1 ( 0.0%) | 0 ( 0.00%) | 1 ( 0.2%) | 0 ( 0.00%) | 0 ( 0.00%) |
|  | Chronic lymphocytic leukaemia | 1 ( 0.0%) | 0 ( 0.00%) | 1 ( 0.2%) | 0 ( 0.00%) | 0 ( 0.00%) |
|  | Colon cancer metastatic | 1 ( 0.0%) | 0 ( 0.00%) | 1 ( 0.2%) | 0 ( 0.00%) | 0 ( 0.00%) |
|  | Gastric cancer | 1 ( 0.0%) | 0 ( 0.00%) | 0 ( 0.00%) | 0 ( 0.00%) | 1 ( 0.5%) |
|  | Hepatic cancer | 1 ( 0.0%) | 0 ( 0.00%) | 1 ( 0.2%) | 0 ( 0.00%) | 0 ( 0.00%) |
|  | Hepatic neoplasm | 1 ( 0.0%) | 0 ( 0.00%) | 1 ( 0.2%) | 0 ( 0.00%) | 0 ( 0.00%) |
|  | Laryngeal cancer | 1 ( 0.0%) | 0 ( 0.00%) | 1 ( 0.2%) | 0 ( 0.00%) | 0 ( 0.00%) |
|  | Malignant neoplasm progression | 1 ( 0.0%) | 0 ( 0.00%) | 1 ( 0.2%) | 0 ( 0.00%) | 0 ( 0.00%) |
|  | Meningioma | 1 ( 0.0%) | 0 ( 0.00%) | 1 ( 0.2%) | 0 ( 0.00%) | 0 ( 0.00%) |
|  | Metastases to bone | 1 ( 0.0%) | 0 ( 0.00%) | 1 ( 0.2%) | 0 ( 0.00%) | 0 ( 0.00%) |
|  | Metastases to lung | 1 ( 0.0%) | 0 ( 0.00%) | 1 ( 0.2%) | 0 ( 0.00%) | 0 ( 0.00%) |
|  | Metastases to lymph nodes | 1 ( 0.0%) | 0 ( 0.00%) | 1 ( 0.2%) | 0 ( 0.00%) | 0 ( 0.00%) |
|  | Metastasis | 1 ( 0.0%) | 0 ( 0.00%) | 1 ( 0.2%) | 0 ( 0.00%) | 0 ( 0.00%) |
|  | Metastatic bronchial carcinoma | 1 ( 0.0%) | 0 ( 0.00%) | 1 ( 0.2%) | 0 ( 0.00%) | 0 ( 0.00%) |
|  | Non-Hodgkin's lymphoma | 1 ( 0.0%) | 0 ( 0.00%) | 1 ( 0.2%) | 0 ( 0.00%) | 0 ( 0.00%) |
|  | Oesophageal carcinoma | 1 ( 0.0%) | 0 ( 0.00%) | 1 ( 0.2%) | 0 ( 0.00%) | 0 ( 0.00%) |
|  | Oligodendroglioma | 1 ( 0.0%) | 0 ( 0.00%) | 1 ( 0.2%) | 0 ( 0.00%) | 0 ( 0.00%) |
|  | Pancreatic carcinoma | 1 ( 0.0%) | 0 ( 0.00%) | 1 ( 0.2%) | 0 ( 0.00%) | 0 ( 0.00%) |
|  | Plasma cell myeloma | 1 ( 0.0%) | 0 ( 0.00%) | 1 ( 0.2%) | 0 ( 0.00%) | 0 ( 0.00%) |
|  | Renal cancer | 1 ( 0.0%) | 0 ( 0.00%) | 1 ( 0.2%) | 0 ( 0.00%) | 0 ( 0.00%) |
|  | Squamous cell carcinoma | 1 ( 0.0%) | 0 ( 0.00%) | 1 ( 0.2%) | 0 ( 0.00%) | 0 ( 0.00%) |
|  | Uterine cancer | 1 ( 0.0%) | 0 ( 0.00%) | 1 ( 0.2%) | 0 ( 0.00%) | 0 ( 0.00%) |
|  | Vulval cancer | 1 ( 0.0%) | 0 ( 0.00%) | 1 ( 0.2%) | 0 ( 0.00%) | 0 ( 0.00%) |
|  |  |  |  |  |  |  |
| Vascular disorders | All Patients | 45 ( 0.8%) | 11 ( 2.4%) | 19 ( 4.6%) | 6 ( 1.2%) | 10 ( 4.7%) |
|  | Hypertensive crisis | 9 ( 0.2%) | 0 ( 0.00%) | 5 ( 1.2%) | 0 ( 0.00%) | 4 ( 1.9%) |
|  | Hypertension | 7 ( 0.1%) | 3 ( 0.7%) | 0 ( 0.00%) | 3 ( 0.6%) | 1 ( 0.5%) |
|  | Neovascularisation | 3 ( 0.1%) | 2 ( 0.4%) | 1 ( 0.2%) | 0 ( 0.00%) | 0 ( 0.00%) |
|  | Thrombosis | 3 ( 0.1%) | 0 ( 0.00%) | 0 ( 0.00%) | 1 ( 0.2%) | 2 ( 0.9%) |
|  | Aortic aneurysm | 2 ( 0.0%) | 0 ( 0.00%) | 0 ( 0.00%) | 1 ( 0.2%) | 1 ( 0.5%) |
|  | Blood pressure fluctuation | 2 ( 0.0%) | 2 ( 0.4%) | 0 ( 0.00%) | 0 ( 0.00%) | 0 ( 0.00%) |
|  | Circulatory collapse | 2 ( 0.0%) | 0 ( 0.00%) | 2 ( 0.5%) | 0 ( 0.00%) | 0 ( 0.00%) |
|  | Deep vein thrombosis | 2 ( 0.0%) | 1 ( 0.2%) | 1 ( 0.2%) | 0 ( 0.00%) | 0 ( 0.00%) |
|  | Pallor | 2 ( 0.0%) | 0 ( 0.00%) | 1 ( 0.2%) | 0 ( 0.00%) | 1 ( 0.5%) |
|  | Peripheral arterial occlusive disease | 2 ( 0.0%) | 1 ( 0.2%) | 1 ( 0.2%) | 0 ( 0.00%) | 0 ( 0.00%) |
|  | Aneurysm | 1 ( 0.0%) | 0 ( 0.00%) | 1 ( 0.2%) | 0 ( 0.00%) | 0 ( 0.00%) |
|  | Arteriosclerosis | 1 ( 0.0%) | 1 ( 0.2%) | 0 ( 0.00%) | 0 ( 0.00%) | 0 ( 0.00%) |
|  | Blood pressure inadequately controlled | 1 ( 0.0%) | 0 ( 0.00%) | 1 ( 0.2%) | 0 ( 0.00%) | 0 ( 0.00%) |
|  | Diabetic microangiopathy | 1 ( 0.0%) | 0 ( 0.00%) | 1 ( 0.2%) | 0 ( 0.00%) | 0 ( 0.00%) |
|  | Embolism | 1 ( 0.0%) | 0 ( 0.00%) | 1 ( 0.2%) | 0 ( 0.00%) | 0 ( 0.00%) |
|  | Haematoma | 1 ( 0.0%) | 0 ( 0.00%) | 1 ( 0.2%) | 0 ( 0.00%) | 0 ( 0.00%) |
|  | Haemorrhage | 1 ( 0.0%) | 1 ( 0.2%) | 0 ( 0.00%) | 0 ( 0.00%) | 0 ( 0.00%) |
|  | Microangiopathy | 1 ( 0.0%) | 0 ( 0.00%) | 0 ( 0.00%) | 1 ( 0.2%) | 0 ( 0.00%) |
|  | Pelvic venous thrombosis | 1 ( 0.0%) | 0 ( 0.00%) | 1 ( 0.2%) | 0 ( 0.00%) | 0 ( 0.00%) |
|  | Poor peripheral circulation | 1 ( 0.0%) | 0 ( 0.00%) | 1 ( 0.2%) | 0 ( 0.00%) | 0 ( 0.00%) |
|  | Varicose vein | 1 ( 0.0%) | 0 ( 0.00%) | 1 ( 0.2%) | 0 ( 0.00%) | 0 ( 0.00%) |
|  | Venous thrombosis | 1 ( 0.0%) | 0 ( 0.00%) | 0 ( 0.00%) | 0 ( 0.00%) | 1 ( 0.5%) |
|  |  |  |  |  |  |  |
| Musculoskeletal and connective tissue disorders | All Patients | 37 ( 0.6%) | 14 ( 3.1%) | 18 ( 4.3%) | 3 ( 0.6%) | 3 ( 1.4%) |
|  | Osteoarthritis | 8 ( 0.1%) | 0 ( 0.00%) | 6 ( 1.4%) | 1 ( 0.2%) | 1 ( 0.5%) |
|  | Pain in extremity | 5 ( 0.1%) | 3 ( 0.7%) | 1 ( 0.2%) | 0 ( 0.00%) | 1 ( 0.5%) |
|  | Sjogren's syndrome | 5 ( 0.1%) | 3 ( 0.7%) | 1 ( 0.2%) | 1 ( 0.2%) | 0 ( 0.00%) |
|  | Arthralgia | 4 ( 0.1%) | 3 ( 0.7%) | 1 ( 0.2%) | 0 ( 0.00%) | 0 ( 0.00%) |
|  | Back pain | 3 ( 0.1%) | 1 ( 0.2%) | 2 ( 0.5%) | 0 ( 0.00%) | 0 ( 0.00%) |
|  | Bone pain | 2 ( 0.0%) | 2 ( 0.4%) | 0 ( 0.00%) | 0 ( 0.00%) | 0 ( 0.00%) |
|  | Musculoskeletal pain | 2 ( 0.0%) | 0 ( 0.00%) | 1 ( 0.2%) | 1 ( 0.2%) | 0 ( 0.00%) |
|  | Spinal column stenosis | 2 ( 0.0%) | 0 ( 0.00%) | 2 ( 0.5%) | 0 ( 0.00%) | 0 ( 0.00%) |
|  | Arthritis | 1 ( 0.0%) | 1 ( 0.2%) | 0 ( 0.00%) | 0 ( 0.00%) | 0 ( 0.00%) |
|  | Arthropathy | 1 ( 0.0%) | 0 ( 0.00%) | 1 ( 0.2%) | 0 ( 0.00%) | 0 ( 0.00%) |
|  | Bursitis | 1 ( 0.0%) | 0 ( 0.00%) | 1 ( 0.2%) | 0 ( 0.00%) | 0 ( 0.00%) |
|  | Intervertebral disc protrusion | 1 ( 0.0%) | 0 ( 0.00%) | 1 ( 0.2%) | 0 ( 0.00%) | 0 ( 0.00%) |
|  | Limb discomfort | 1 ( 0.0%) | 1 ( 0.2%) | 0 ( 0.00%) | 0 ( 0.00%) | 0 ( 0.00%) |
|  | Mobility decreased | 1 ( 0.0%) | 0 ( 0.00%) | 0 ( 0.00%) | 0 ( 0.00%) | 1 ( 0.5%) |
|  | Muscular weakness | 1 ( 0.0%) | 0 ( 0.00%) | 1 ( 0.2%) | 0 ( 0.00%) | 0 ( 0.00%) |
|  | Musculoskeletal discomfort | 1 ( 0.0%) | 1 ( 0.2%) | 0 ( 0.00%) | 0 ( 0.00%) | 0 ( 0.00%) |
|  | Musculoskeletal stiffness | 1 ( 0.0%) | 0 ( 0.00%) | 1 ( 0.2%) | 0 ( 0.00%) | 0 ( 0.00%) |
|  | Neck pain | 1 ( 0.0%) | 1 ( 0.2%) | 0 ( 0.00%) | 0 ( 0.00%) | 0 ( 0.00%) |
|  | Osteitis | 1 ( 0.0%) | 1 ( 0.2%) | 0 ( 0.00%) | 0 ( 0.00%) | 0 ( 0.00%) |
|  | Pain in jaw | 1 ( 0.0%) | 0 ( 0.00%) | 1 ( 0.2%) | 0 ( 0.00%) | 0 ( 0.00%) |
|  | Rheumatic disorder | 1 ( 0.0%) | 1 ( 0.2%) | 0 ( 0.00%) | 0 ( 0.00%) | 0 ( 0.00%) |
|  |  |  |  |  |  |  |
| Respiratory, thoracic and mediastinal disorders | All Patients | 33 ( 0.6%) | 6 ( 1.3%) | 21 ( 5.0%) | 4 ( 0.8%) | 7 ( 3.3%) |
|  | Dyspnoea | 10 ( 0.2%) | 0 ( 0.00%) | 5 ( 1.2%) | 1 ( 0.2%) | 4 ( 1.9%) |
|  | Cough | 7 ( 0.1%) | 4 ( 0.9%) | 1 ( 0.2%) | 2 ( 0.4%) | 0 ( 0.00%) |
|  | Pulmonary embolism | 7 ( 0.1%) | 0 ( 0.00%) | 5 ( 1.2%) | 0 ( 0.00%) | 2 ( 0.9%) |
|  | Chronic obstructive pulmonary disease | 5 ( 0.1%) | 0 ( 0.00%) | 4 ( 1.0%) | 1 ( 0.2%) | 0 ( 0.00%) |
|  | Pulmonary oedema | 5 ( 0.1%) | 0 ( 0.00%) | 4 ( 1.0%) | 1 ( 0.2%) | 1 ( 0.5%) |
|  | Pleural effusion | 2 ( 0.0%) | 0 ( 0.00%) | 2 ( 0.5%) | 0 ( 0.00%) | 0 ( 0.00%) |
|  | Respiratory failure | 2 ( 0.0%) | 0 ( 0.00%) | 1 ( 0.2%) | 0 ( 0.00%) | 1 ( 0.5%) |
|  | Bronchial disorder | 1 ( 0.0%) | 0 ( 0.00%) | 1 ( 0.2%) | 0 ( 0.00%) | 0 ( 0.00%) |
|  | Diaphragmatic disorder | 1 ( 0.0%) | 0 ( 0.00%) | 0 ( 0.00%) | 1 ( 0.2%) | 0 ( 0.00%) |
|  | Dysphonia | 1 ( 0.0%) | 1 ( 0.2%) | 0 ( 0.00%) | 0 ( 0.00%) | 0 ( 0.00%) |
|  | Dyspnoea at rest | 1 ( 0.0%) | 0 ( 0.00%) | 0 ( 0.00%) | 0 ( 0.00%) | 1 ( 0.5%) |
|  | Epistaxis | 1 ( 0.0%) | 0 ( 0.00%) | 1 ( 0.2%) | 0 ( 0.00%) | 0 ( 0.00%) |
|  | Hypercapnia | 1 ( 0.0%) | 0 ( 0.00%) | 0 ( 0.00%) | 1 ( 0.2%) | 0 ( 0.00%) |
|  | Hypoxia | 1 ( 0.0%) | 0 ( 0.00%) | 1 ( 0.2%) | 0 ( 0.00%) | 0 ( 0.00%) |
|  | Pleurisy | 1 ( 0.0%) | 1 ( 0.2%) | 0 ( 0.00%) | 0 ( 0.00%) | 0 ( 0.00%) |
|  | Pulmonary arterial hypertension | 1 ( 0.0%) | 0 ( 0.00%) | 0 ( 0.00%) | 0 ( 0.00%) | 1 ( 0.5%) |
|  |  |  |  |  |  |  |
| Gastrointestinal disorders | All Patients | 26 ( 0.4%) | 10 ( 2.2%) | 11 ( 2.6%) | 4 ( 0.8%) | 3 ( 1.4%) |
|  | Diarrhoea | 4 ( 0.1%) | 4 ( 0.9%) | 0 ( 0.00%) | 0 ( 0.00%) | 0 ( 0.00%) |
|  | Abdominal pain upper | 3 ( 0.1%) | 3 ( 0.7%) | 0 ( 0.00%) | 0 ( 0.00%) | 0 ( 0.00%) |
|  | Gastritis | 3 ( 0.1%) | 1 ( 0.2%) | 2 ( 0.5%) | 0 ( 0.00%) | 0 ( 0.00%) |
|  | Ascites | 2 ( 0.0%) | 0 ( 0.00%) | 2 ( 0.5%) | 0 ( 0.00%) | 0 ( 0.00%) |
|  | Gastric ulcer | 2 ( 0.0%) | 0 ( 0.00%) | 1 ( 0.2%) | 1 ( 0.2%) | 0 ( 0.00%) |
|  | Nausea | 2 ( 0.0%) | 2 ( 0.4%) | 0 ( 0.00%) | 1 ( 0.2%) | 0 ( 0.00%) |
|  | Pancreatitis | 2 ( 0.0%) | 0 ( 0.00%) | 2 ( 0.5%) | 0 ( 0.00%) | 0 ( 0.00%) |
|  | Vomiting | 2 ( 0.0%) | 0 ( 0.00%) | 1 ( 0.2%) | 1 ( 0.2%) | 0 ( 0.00%) |
|  | Dry mouth | 1 ( 0.0%) | 0 ( 0.00%) | 0 ( 0.00%) | 1 ( 0.2%) | 0 ( 0.00%) |
|  | Duodenal ulcer | 1 ( 0.0%) | 0 ( 0.00%) | 0 ( 0.00%) | 1 ( 0.2%) | 0 ( 0.00%) |
|  | Dysphagia | 1 ( 0.0%) | 0 ( 0.00%) | 1 ( 0.2%) | 0 ( 0.00%) | 0 ( 0.00%) |
|  | Gastrointestinal disorder | 1 ( 0.0%) | 1 ( 0.2%) | 0 ( 0.00%) | 0 ( 0.00%) | 0 ( 0.00%) |
|  | Gastrointestinal haemorrhage | 1 ( 0.0%) | 0 ( 0.00%) | 1 ( 0.2%) | 0 ( 0.00%) | 0 ( 0.00%) |
|  | Gastrointestinal inflammation | 1 ( 0.0%) | 0 ( 0.00%) | 0 ( 0.00%) | 1 ( 0.2%) | 0 ( 0.00%) |
|  | Gastrooesophageal reflux disease | 1 ( 0.0%) | 0 ( 0.00%) | 1 ( 0.2%) | 0 ( 0.00%) | 0 ( 0.00%) |
|  | Haematochezia | 1 ( 0.0%) | 0 ( 0.00%) | 0 ( 0.00%) | 0 ( 0.00%) | 1 ( 0.5%) |
|  | Ileus | 1 ( 0.0%) | 0 ( 0.00%) | 0 ( 0.00%) | 0 ( 0.00%) | 1 ( 0.5%) |
|  | Intestinal haemorrhage | 1 ( 0.0%) | 0 ( 0.00%) | 0 ( 0.00%) | 0 ( 0.00%) | 1 ( 0.5%) |
|  | Intestinal perforation | 1 ( 0.0%) | 0 ( 0.00%) | 1 ( 0.2%) | 0 ( 0.00%) | 0 ( 0.00%) |
|  | Large intestinal stenosis | 1 ( 0.0%) | 0 ( 0.00%) | 1 ( 0.2%) | 0 ( 0.00%) | 0 ( 0.00%) |
|  | Loose tooth | 1 ( 0.0%) | 1 ( 0.2%) | 0 ( 0.00%) | 0 ( 0.00%) | 0 ( 0.00%) |
|  | Volvulus | 1 ( 0.0%) | 0 ( 0.00%) | 0 ( 0.00%) | 0 ( 0.00%) | 1 ( 0.5%) |
|  |  |  |  |  |  |  |
| Metabolism and nutrition disorders | All Patients | 19 ( 0.3%) | 8 ( 1.8%) | 7 ( 1.7%) | 4 ( 0.8%) | 1 ( 0.5%) |
|  | Diabetes mellitus | 3 ( 0.1%) | 1 ( 0.2%) | 2 ( 0.5%) | 0 ( 0.00%) | 0 ( 0.00%) |
|  | Gout | 3 ( 0.1%) | 3 ( 0.7%) | 0 ( 0.00%) | 1 ( 0.2%) | 0 ( 0.00%) |
|  | Hypoglycaemia | 3 ( 0.1%) | 2 ( 0.4%) | 0 ( 0.00%) | 0 ( 0.00%) | 1 ( 0.5%) |
|  | Decreased appetite | 2 ( 0.0%) | 1 ( 0.2%) | 0 ( 0.00%) | 1 ( 0.2%) | 0 ( 0.00%) |
|  | Cachexia | 1 ( 0.0%) | 0 ( 0.00%) | 1 ( 0.2%) | 0 ( 0.00%) | 0 ( 0.00%) |
|  | Diabetic metabolic decompensation | 1 ( 0.0%) | 0 ( 0.00%) | 1 ( 0.2%) | 0 ( 0.00%) | 0 ( 0.00%) |
|  | Hypercholesterolaemia | 1 ( 0.0%) | 1 ( 0.2%) | 0 ( 0.00%) | 0 ( 0.00%) | 0 ( 0.00%) |
|  | Hyperglycaemia | 1 ( 0.0%) | 0 ( 0.00%) | 1 ( 0.2%) | 0 ( 0.00%) | 0 ( 0.00%) |
|  | Hyperlipidaemia | 1 ( 0.0%) | 0 ( 0.00%) | 0 ( 0.00%) | 1 ( 0.2%) | 0 ( 0.00%) |
|  | Hypernatraemia | 1 ( 0.0%) | 0 ( 0.00%) | 1 ( 0.2%) | 0 ( 0.00%) | 0 ( 0.00%) |
|  | Insulin resistance | 1 ( 0.0%) | 1 ( 0.2%) | 0 ( 0.00%) | 0 ( 0.00%) | 0 ( 0.00%) |
|  | Metabolic acidosis | 1 ( 0.0%) | 0 ( 0.00%) | 0 ( 0.00%) | 1 ( 0.2%) | 0 ( 0.00%) |
|  | Metabolic disorder | 1 ( 0.0%) | 0 ( 0.00%) | 1 ( 0.2%) | 0 ( 0.00%) | 0 ( 0.00%) |
|  | Underweight | 1 ( 0.0%) | 0 ( 0.00%) | 1 ( 0.2%) | 0 ( 0.00%) | 0 ( 0.00%) |
|  |  |  |  |  |  |  |
| Skin and subcutaneous tissue disorders | All Patients | 16 ( 0.3%) | 6 ( 1.3%) | 2 ( 0.5%) | 8 ( 1.6%) | 1 ( 0.5%) |
|  | Pruritus | 5 ( 0.1%) | 2 ( 0.4%) | 0 ( 0.00%) | 3 ( 0.6%) | 0 ( 0.00%) |
|  | Urticaria | 3 ( 0.1%) | 0 ( 0.00%) | 0 ( 0.00%) | 3 ( 0.6%) | 0 ( 0.00%) |
|  | Rash | 2 ( 0.0%) | 1 ( 0.2%) | 0 ( 0.00%) | 1 ( 0.2%) | 0 ( 0.00%) |
|  | Acne | 1 ( 0.0%) | 0 ( 0.00%) | 0 ( 0.00%) | 1 ( 0.2%) | 0 ( 0.00%) |
|  | Actinic keratosis | 1 ( 0.0%) | 1 ( 0.2%) | 0 ( 0.00%) | 0 ( 0.00%) | 0 ( 0.00%) |
|  | Cold sweat | 1 ( 0.0%) | 0 ( 0.00%) | 1 ( 0.2%) | 0 ( 0.00%) | 0 ( 0.00%) |
|  | Dermatitis allergic | 1 ( 0.0%) | 0 ( 0.00%) | 0 ( 0.00%) | 1 ( 0.2%) | 0 ( 0.00%) |
|  | Diabetic foot | 1 ( 0.0%) | 0 ( 0.00%) | 1 ( 0.2%) | 0 ( 0.00%) | 0 ( 0.00%) |
|  | Eczema nummular | 1 ( 0.0%) | 0 ( 0.00%) | 0 ( 0.00%) | 1 ( 0.2%) | 0 ( 0.00%) |
|  | Erythema | 1 ( 0.0%) | 0 ( 0.00%) | 0 ( 0.00%) | 0 ( 0.00%) | 1 ( 0.5%) |
|  | Fixed drug eruption | 1 ( 0.0%) | 0 ( 0.00%) | 0 ( 0.00%) | 1 ( 0.2%) | 0 ( 0.00%) |
|  | Hyperhidrosis | 1 ( 0.0%) | 0 ( 0.00%) | 0 ( 0.00%) | 1 ( 0.2%) | 0 ( 0.00%) |
|  | Nail bed inflammation | 1 ( 0.0%) | 1 ( 0.2%) | 0 ( 0.00%) | 0 ( 0.00%) | 0 ( 0.00%) |
|  | Papule | 1 ( 0.0%) | 0 ( 0.00%) | 0 ( 0.00%) | 1 ( 0.2%) | 0 ( 0.00%) |
|  | Pruritus generalised | 1 ( 0.0%) | 1 ( 0.2%) | 0 ( 0.00%) | 0 ( 0.00%) | 0 ( 0.00%) |
|  | Rash erythematous | 1 ( 0.0%) | 0 ( 0.00%) | 0 ( 0.00%) | 1 ( 0.2%) | 0 ( 0.00%) |
|  | Rosacea | 1 ( 0.0%) | 1 ( 0.2%) | 0 ( 0.00%) | 0 ( 0.00%) | 0 ( 0.00%) |
|  | Skin oedema | 1 ( 0.0%) | 0 ( 0.00%) | 0 ( 0.00%) | 1 ( 0.2%) | 0 ( 0.00%) |
|  | Skin swelling | 1 ( 0.0%) | 0 ( 0.00%) | 0 ( 0.00%) | 1 ( 0.2%) | 0 ( 0.00%) |
|  | Xanthelasma | 1 ( 0.0%) | 1 ( 0.2%) | 0 ( 0.00%) | 0 ( 0.00%) | 0 ( 0.00%) |
|  |  |  |  |  |  |  |
| Renal and urinary disorders | All Patients | 12 ( 0.2%) | 1 ( 0.2%) | 5 ( 1.2%) | 2 ( 0.4%) | 4 ( 1.9%) |
|  | Renal failure | 4 ( 0.1%) | 0 ( 0.00%) | 2 ( 0.5%) | 0 ( 0.00%) | 2 ( 0.9%) |
|  | Chronic kidney disease | 2 ( 0.0%) | 1 ( 0.2%) | 1 ( 0.2%) | 0 ( 0.00%) | 0 ( 0.00%) |
|  | Cystitis noninfective | 1 ( 0.0%) | 0 ( 0.00%) | 0 ( 0.00%) | 1 ( 0.2%) | 0 ( 0.00%) |
|  | Haematuria | 1 ( 0.0%) | 0 ( 0.00%) | 1 ( 0.2%) | 0 ( 0.00%) | 0 ( 0.00%) |
|  | Nephropathy | 1 ( 0.0%) | 0 ( 0.00%) | 1 ( 0.2%) | 0 ( 0.00%) | 0 ( 0.00%) |
|  | Renal artery stenosis | 1 ( 0.0%) | 0 ( 0.00%) | 0 ( 0.00%) | 0 ( 0.00%) | 1 ( 0.5%) |
|  | Renal cyst | 1 ( 0.0%) | 0 ( 0.00%) | 0 ( 0.00%) | 0 ( 0.00%) | 1 ( 0.5%) |
|  | Renal disorder | 1 ( 0.0%) | 0 ( 0.00%) | 0 ( 0.00%) | 1 ( 0.2%) | 0 ( 0.00%) |
|  | Renal embolism | 1 ( 0.0%) | 0 ( 0.00%) | 0 ( 0.00%) | 0 ( 0.00%) | 1 ( 0.5%) |
|  | Tubulointerstitial nephritis | 1 ( 0.0%) | 0 ( 0.00%) | 1 ( 0.2%) | 0 ( 0.00%) | 0 ( 0.00%) |
|  | Urethral stenosis | 1 ( 0.0%) | 0 ( 0.00%) | 1 ( 0.2%) | 0 ( 0.00%) | 0 ( 0.00%) |
|  |  |  |  |  |  |  |
| Psychiatric disorders | All Patients | 11 ( 0.2%) | 4 ( 0.9%) | 6 ( 1.4%) | 0 ( 0.00%) | 1 ( 0.5%) |
|  | Depression | 5 ( 0.1%) | 3 ( 0.7%) | 2 ( 0.5%) | 0 ( 0.00%) | 0 ( 0.00%) |
|  | Confusional state | 2 ( 0.0%) | 0 ( 0.00%) | 1 ( 0.2%) | 0 ( 0.00%) | 1 ( 0.5%) |
|  | Agitation | 1 ( 0.0%) | 1 ( 0.2%) | 0 ( 0.00%) | 0 ( 0.00%) | 0 ( 0.00%) |
|  | Alcohol abuse | 1 ( 0.0%) | 0 ( 0.00%) | 1 ( 0.2%) | 0 ( 0.00%) | 0 ( 0.00%) |
|  | Completed suicide | 1 ( 0.0%) | 0 ( 0.00%) | 1 ( 0.2%) | 0 ( 0.00%) | 0 ( 0.00%) |
|  | Disorientation | 1 ( 0.0%) | 0 ( 0.00%) | 0 ( 0.00%) | 0 ( 0.00%) | 1 ( 0.5%) |
|  | Restlessness | 1 ( 0.0%) | 0 ( 0.00%) | 1 ( 0.2%) | 0 ( 0.00%) | 0 ( 0.00%) |
|  |  |  |  |  |  |  |
| Hepatobiliary disorders | All Patients | 9 ( 0.2%) | 0 ( 0.00%) | 8 ( 1.9%) | 0 ( 0.00%) | 1 ( 0.5%) |
|  | Cholelithiasis | 4 ( 0.1%) | 0 ( 0.00%) | 4 ( 1.0%) | 0 ( 0.00%) | 0 ( 0.00%) |
|  | Cholecystitis | 2 ( 0.0%) | 0 ( 0.00%) | 2 ( 0.5%) | 0 ( 0.00%) | 0 ( 0.00%) |
|  | Liver disorder | 2 ( 0.0%) | 0 ( 0.00%) | 2 ( 0.5%) | 0 ( 0.00%) | 0 ( 0.00%) |
|  | Bile duct obstruction | 1 ( 0.0%) | 0 ( 0.00%) | 0 ( 0.00%) | 0 ( 0.00%) | 1 ( 0.5%) |
|  | Biliary colic | 1 ( 0.0%) | 0 ( 0.00%) | 1 ( 0.2%) | 0 ( 0.00%) | 0 ( 0.00%) |
|  |  |  |  |  |  |  |
| Ear and labyrinth disorders | All Patients | 8 ( 0.1%) | 5 ( 1.1%) | 2 ( 0.5%) | 1 ( 0.2%) | 0 ( 0.00%) |
|  | Vertigo | 6 ( 0.1%) | 4 ( 0.9%) | 1 ( 0.2%) | 1 ( 0.2%) | 0 ( 0.00%) |
|  | Hypoacusis | 1 ( 0.0%) | 1 ( 0.2%) | 0 ( 0.00%) | 0 ( 0.00%) | 0 ( 0.00%) |
|  | Tinnitus | 1 ( 0.0%) | 0 ( 0.00%) | 1 ( 0.2%) | 0 ( 0.00%) | 0 ( 0.00%) |
|  |  |  |  |  |  |  |
| Blood and lymphatic system disorders | All Patients | 7 ( 0.1%) | 1 ( 0.2%) | 5 ( 1.2%) | 0 ( 0.00%) | 1 ( 0.5%) |
|  | Anaemia | 3 ( 0.1%) | 0 ( 0.00%) | 3 ( 0.7%) | 0 ( 0.00%) | 0 ( 0.00%) |
|  | Iron deficiency anaemia | 2 ( 0.0%) | 1 ( 0.2%) | 1 ( 0.2%) | 0 ( 0.00%) | 0 ( 0.00%) |
|  | Polycythaemia | 1 ( 0.0%) | 0 ( 0.00%) | 0 ( 0.00%) | 0 ( 0.00%) | 1 ( 0.5%) |
|  | Thrombocytosis | 1 ( 0.0%) | 0 ( 0.00%) | 1 ( 0.2%) | 0 ( 0.00%) | 0 ( 0.00%) |
|  |  |  |  |  |  |  |
| Immune system disorders | All Patients | 5 ( 0.1%) | 3 ( 0.7%) | 1 ( 0.2%) | 1 ( 0.2%) | 0 ( 0.00%) |
|  | Allergy to chemicals | 1 ( 0.0%) | 0 ( 0.00%) | 0 ( 0.00%) | 1 ( 0.2%) | 0 ( 0.00%) |
|  | Hypersensitivity | 1 ( 0.0%) | 1 ( 0.2%) | 0 ( 0.00%) | 0 ( 0.00%) | 0 ( 0.00%) |
|  | Sarcoidosis | 1 ( 0.0%) | 0 ( 0.00%) | 1 ( 0.2%) | 0 ( 0.00%) | 0 ( 0.00%) |
|  | Seasonal allergy | 1 ( 0.0%) | 1 ( 0.2%) | 0 ( 0.00%) | 0 ( 0.00%) | 0 ( 0.00%) |
|  | Type IV hypersensitivity reaction | 1 ( 0.0%) | 1 ( 0.2%) | 0 ( 0.00%) | 0 ( 0.00%) | 0 ( 0.00%) |
| Social circumstances | All Patients | 5 ( 0.1%) | 2 ( 0.4%) | 2 ( 0.5%) | 1 ( 0.2%) | 0 ( 0.00%) |
|  | Refusal of treatment by patient | 2 ( 0.0%) | 2 ( 0.4%) | 0 ( 0.00%) | 0 ( 0.00%) | 0 ( 0.00%) |
|  | Death of relative | 1 ( 0.0%) | 0 ( 0.00%) | 0 ( 0.00%) | 1 ( 0.2%) | 0 ( 0.00%) |
|  | Device dependence | 1 ( 0.0%) | 0 ( 0.00%) | 1 ( 0.2%) | 0 ( 0.00%) | 0 ( 0.00%) |
|  | Walking disability | 1 ( 0.0%) | 0 ( 0.00%) | 1 ( 0.2%) | 0 ( 0.00%) | 0 ( 0.00%) |
|  |  |  |  |  |  |  |
| Not codable | Not codable | 3 ( 0.1%) | 0 ( 0.00%) | 0 ( 0.00%) | 2 ( 0.4%) | 1 ( 0.5%) |
|  | All Patients | 3 ( 0.1%) | 0 ( 0.00%) | 0 ( 0.00%) | 2 ( 0.4%) | 1 ( 0.5%) |
|  |  |  |  |  |  |  |
| Congenital, familial and genetic disorders | All Patients | 3 ( 0.1%) | 0 ( 0.00%) | 1 ( 0.2%) | 0 ( 0.00%) | 2 ( 0.9%) |
|  | Hypertrophic cardiomyopathy | 2 ( 0.0%) | 0 ( 0.00%) | 1 ( 0.2%) | 0 ( 0.00%) | 1 ( 0.5%) |
|  | Atrial septal defect | 1 ( 0.0%) | 0 ( 0.00%) | 0 ( 0.00%) | 0 ( 0.00%) | 1 ( 0.5%) |
|  |  |  |  |  |  |  |
| Endocrine disorders | All Patients | 3 ( 0.1%) | 2 ( 0.4%) | 0 ( 0.00%) | 1 ( 0.2%) | 0 ( 0.00%) |
|  | Hyperthyroidism | 3 ( 0.1%) | 2 ( 0.4%) | 0 ( 0.00%) | 1 ( 0.2%) | 0 ( 0.00%) |
|  |  |  |  |  |  |  |
| Product issues | All Patients | 3 ( 0.1%) | 1 ( 0.2%) | 2 ( 0.5%) | 0 ( 0.00%) | 0 ( 0.00%) |
|  | Device breakage | 1 ( 0.0%) | 1 ( 0.2%) | 0 ( 0.00%) | 0 ( 0.00%) | 0 ( 0.00%) |
|  | Device failure | 1 ( 0.0%) | 0 ( 0.00%) | 1 ( 0.2%) | 0 ( 0.00%) | 0 ( 0.00%) |
|  | Device material opacification | 1 ( 0.0%) | 0 ( 0.00%) | 1 ( 0.2%) | 0 ( 0.00%) | 0 ( 0.00%) |
|  |  |  |  |  |  |  |
| Reproductive system and breast disorders | All Patients | 2 ( 0.0%) | 0 ( 0.00%) | 2 ( 0.5%) | 0 ( 0.00%) | 0 ( 0.00%) |
|  | Cervical polyp | 1 ( 0.0%) | 0 ( 0.00%) | 1 ( 0.2%) | 0 ( 0.00%) | 0 ( 0.00%) |
|  | Prostatic disorder | 1 ( 0.0%) | 0 ( 0.00%) | 1 ( 0.2%) | 0 ( 0.00%) | 0 ( 0.00%) |
